# Main Habitat Factors Driving the Phenotypic Diversity of *Litsea cubeba* in China

**DOI:** 10.3390/plants12213781

**Published:** 2023-11-06

**Authors:** Guoxiang Liao, Xiaodan Ning, Yuling Yang, Zongde Wang, Guorong Fan, Xuefang Wang, Dan Fu, Juan Liu, Ming Tang, Shangxing Chen, Jiawei Wang

**Affiliations:** 1 Jiangxi Key Laboratory of Silviculture, College of Forestry, Jiangxi Agricultural University, Nanchang 330045, China; lindata183@stu.jxau.edu.cn (G.L.); xiaodanning2021@stu.jxau.edu.cn (X.N.);; 2 East China Woody Fragrance and Flavor Engineering Research Center of National Forestry and Grassland Administration, College of Forestry, Jiangxi Agricultural University, Nanchang 330045, China

**Keywords:** morphological diversity, leaf anatomy, essential oils, geographic distribution

## Abstract

*Litsea cubeba* (Lour.) Pers. is an important woody spice tree in southern China, and its fruit is a rich source of valuable essential oil. We surveyed and sampled *L. cubeba* germplasm resources from 36 provenances in nine Chinese provinces, and detected rich phenotypic diversity. The survey results showed that plants of SC-KJ, SC-HJ, and SC-LS provenance presented higher leaf area (LA); YN-SM and YN-XC plants had larger thousand-grain fresh weight (TFW); and HN-DX plants had the highest essential oil content (EOC). To explain the large differences in the phenotypes of *L. cubeba* among different habitats, we used Pearson’s correlation analysis, multiple stepwise regression path analysis, and redundancy analysis to evaluate the phenotypic diversity of *L. cubeba*. It was found that compared to other traits, leaf and fruit traits had more significant geographical distributions, and that leaf phenotypes were correlated to fruit phenotypes. The results showed that elevation, latitude, longitude, total soil porosity (SP), soil bulk density (SBD), and average annual rainfall (AAR, mm) contributed significantly to the phenotypic diversity of *L. cubeba*. Geographical factors explained a higher percentage of variation in phenotypic diversity than did soil factors and climate factors. Plants of SC-KJ and HN-DX provenances could be important resources for domestication and breeding to develop new high-yielding varieties of this woody aromatic plant. This study describes significant phenotypic differences in *L. cubeba* related to adaptation to different environments, and provides a theoretical basis for the development of a breeding strategy and for optimizing *L. cubeba* cultivation.

## 1. Introduction

*Litsea cubeba* (Lour.) Pers. is a perennial deciduous shrub or small tree belonging to the family Lauraceae [1,2]. It is widely distributed in southern China and other areas of Southeast Asia [3,4]. The plant as a whole has a unique fragrance, but the essential oil is extracted mainly from the fruit [5]. The oil is used in a variety of applications, and the fruit is a common herbal medicine [6]. *L. cubeba* fruits are rich in natural aromatic compounds, and the essential oil extracted from the fruit has a pleasant aroma as well as broad-spectrum antimicrobial properties [7,8] and antioxidant activity [9]. Because *L. cubeba* essential oil has a high economic value, it is highly sought after in domestic and international markets and has attracted the attention of researchers. In a recent survey of the germplasm resources of *L. cubeba*, we found rich phenotypic diversity and relatively low utilization rates [10,11].

Plant phenotypes result from genetic and environmental interactions [12,13]. At the genetic level, genes affect protein expression, which influences phenotype [14]. Genetic factors determine plant phenotypic traits, including growth status [15], leaf morphology [16], fruit morphology [17], and metabolite profile [18]. Environmental factors can also affect plant phenotypes. For example, trait differences among populations in different geographical environments have been detected in *Olea europaea* [19], *Phoenix canariensis* [20], and *Prunus armeniaca* [21]. Previous studies have shown that nutrient stress has a significant effect on the macroscopic characteristics of *Arabidopsis* [22]. Another study reported that *Quercus faginea* populations show altered phenotypic characters such as more sclerotia and lower growth rates when growing in harsher (drier and colder) environments [23].

Recent studies on *L. cubeba* have included ecological resource surveys [24], technical studies on seedling propagation [1], analyses of the antibacterial and antioxidant effects of essential oil [25], and the use of molecular biology methods to determine the mechanism of terpene metabolism with a view to improve terpenoid yield [26]. Relatively few studies have explored how geographical features, climatic conditions, and soil physicochemical properties affect traits such as the fruit yield (FY), essential oil content (EOC), and citral content of *L. cubeba* [10]. Breeding work using elite *L. cubeba* families has been carried out, but it is difficult to breed new varieties in a short period of time [27]. In actual production, there is a scarcity of good *L. cubeba* varieties, and this limits the development of the industry [11]. A more comprehensive survey would reflect the real situation of wild *L. cubeba* populations, allow for the selection and breeding of high-quality *L. cubeba* lines, and provide information to develop optimal cultivation strategies.

At present, wild populations of *L. cubeba* are facing the loss of germplasm resources. It is necessary to comprehensively investigate and clarify the diversity of natural populations to devise appropriate strategies for their protection and use. A few studies have explored how geographical features and climatic conditions affect traits such as FY, EOC, and citral content [14]. Fan et al. (2023) [10] found that FY and EOC are greatly affected by the environment. They found that the EOC decreases and the citral content increases with increasing longitude. A study on the relationship between fruit quality and five environmental factors detected a strong relationship between EOC and precipitation. A more comprehensive study will provide more complete information about the environmental factors in the natural distribution range of wild *L. cubeba* and about the phenotypic diversity of *L. cubeba* in natural populations, and reveal which environmental factors are drivers of this diversity. In this study, we conducted an extensive survey and collected samples across the distribution area of *L. cubeba* in China. We collected 750 trees from 36 provenances in nine provinces. The aim was to explore the phenotypic diversity of *L. cubeba* and to reveal the intrinsic relationship between phenotypic traits and environmental factors. This information will be useful to identify high-quality germplasm resources and provide a reference for the breeding of *L. cubeba* and the cultivation of this shrub on a commercial scale.

## 2. Results

### 2.1. Main Phenotypic Diversity Characteristics of L. cubeba

**Figure 1:**
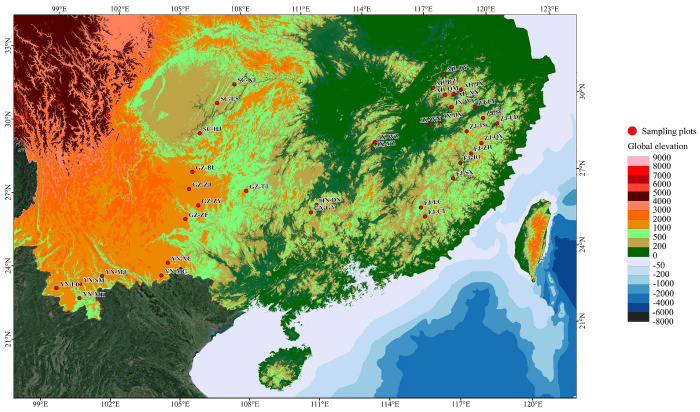
Schematic diagram of sampling of 36 provenances from nine provinces in southern China.

We investigated *L. cubeba* communities represented by 36 provenances in the main distribution areas of *L. cubeba* in nine Chinese provinces and autonomous regions, as shown in Figure 1. The provenances investigated covered the natural distribution areas of *L. cubeba* in China, and the randomly selected provenances were a good representation of the local *L. cubeba* communities’ characteristics. The germplasm resource survey covered a wide latitudinal and longitudinal range, spanning from 22.01 °N to 30.88 °N and from 99.18 °E to 119.99 °E. The elevation of the sites ranged from 13 m to 2014 m, and approximately 76.4% (573 trees) of resources were distributed in hilly or mountainous terrain.

The survey of *L. cubeba* germplasm resources revealed rich phenotypic diversity. We detected a wide range of variation in the traits of *L. cubeba*. Except for fresh fruit pulp rate (FFPR) and leaf shape index (LSI, leaf length/leaf width), the other phenotypic characters showed large differences between the minimum and maximum values. In particular, the maximum values of fruit yield/Basal diameter (FY/BD) were more than 2148 times the minimum values (Table S1). The CV of 20 phenotypes of *L. cubeba* ranged from 7.15% (FFPR) to 218.84% (FY). The CVs were lower for FFPR and water content of fruit (FWC) than for other characteristics (Table S2), indicating that the phenotypic characteristics of the fruit were stable. In addition, the CV of the 36 provenances ranged from 23.13% (ZJ-QY) to 59.76% (YN-XC), with an average of 35.87% (Table S3), indicating a high degree of phenotypic variation of *L. cubeba* at the population level. The average CVs of FFPR and FWC among the 20 phenotypic characteristics were lower than 10%, indicating that the fruit phenotype was relatively stable and consistent with the results shown in Table S2. Of the 20 phenotypes of 36 *L. cubeba* provenances, tree height (TH), TFW, thousand-grain dry weight (TDW), petiole length (PL), FWC, FFPR, leaf length (LL, distance from base to apex), and EOC showed the highest diversity (H ≥ 2.00), while FY showed the lowest diversity among populations (Table S5, H = 0.79). The H′ value has a certain relationship with sample size. The sample sizes for the JX-DX, JX-WY, YN-LC, and AH-QY provenances were relatively small, and all these provenances had low H′ values, consistent with the results in Table S1. This result highlighted the importance of selecting appropriate sample sizes when investigating germplasm resources. Nested ANOVA showed that the 20 phenotypes differed significantly among populations. Some growth characteristics (basal diameter (BD, diameter at ground level), crown diameter (CD), and TH), leaf phenotypes (leaf perimeter (LP), PL and LSI), essential oil yield/Basal diameter (EOY/BD), FY and FY/BD also showed significant differences within populations (Table S4).

**Figure 2:**
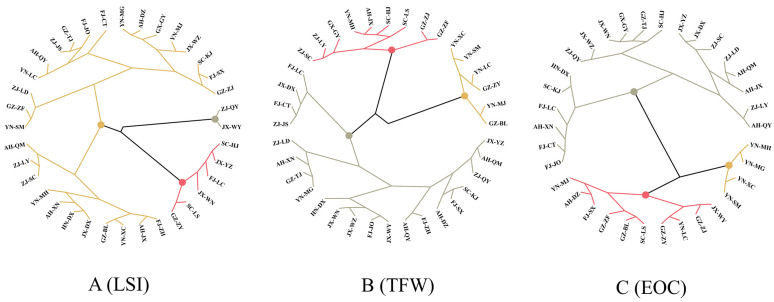
Cluster analyses of 36 provenances of *L. cubeba* on the basis of LSI, TFW and EOC. Hierarchical clustering analysis of 36 provenances based on LSI (**A**), TFW (**B**) and EOC (**C**). Groups of provenances showed in different colors.

The distribution of trait values of *L. cubeba* did not show any clear regional pattern. The average LSI was 3.38 across all provenances. The LSI ranged from 2.55 (SC-LS) to 4.44 (ZJ-QY), and was also high in SC-KJ (4.17). The 36 provenances formed three groups in the hierarchical clustering analysis based on LSI (Figure 2A): group 1 contained ZJ-QY and JX-WY with the highest LSI; group 3 contained the provenances with the lowest LSI (2.55–2.94); and group 2 contained the remaining 28 provenances with intermediate LSI (3.02–3.79). The average TFW was 131.57 g across all provenances. The TFW ranged from 99.18 g in FJ-LC to 178.86 g in GZ-BL. The 34 provenances formed four groups in the hierarchical clustering analysis based on TFW (Figure 2B). Group 1 consisting of GZ-BL, YN-MJ, YN-LC, YN-SM, and YN-XC with the highest TFW. The groups were ranked, from highest TFW to lowest, as follows: group 1 (153.18–178.86 g), group 2 (133.94–146.74 g), and group 3 (99.18–128.04 g). The average EOC was 3.07% across all provenances. The EOC ranged from 1.79% in YN-MH to 4.22% in HN-DX, and was also high in SC-KJ (4.17%). The 34 provenances formed four groups in the hierarchical clustering analysis based on EOC (Figure 2C). Group 3 consisted of YN-SM, YN-XC, YN-MG, and YN-MH with the lowest EOC. The groups were ranked, from highest EOC to lowest, as follows: group 1 (3.00–4.22%), group 2 (2.44–2.86%), group 3 (1.75–2.24%).

### 2.2. Correlations between Phenotypes

**Figure 3:**
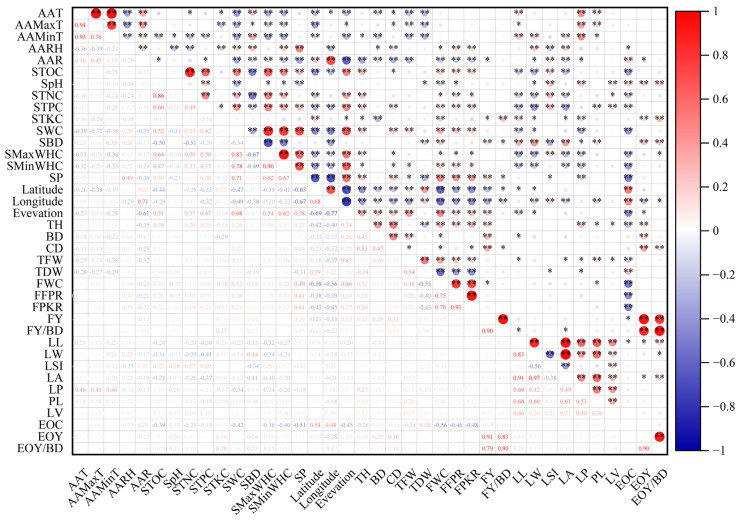
Correlation analysis between environmental factors and phenotypes among provenances. Asterisks indicate significance level (* *p* ≤ 0.05 and ** *p* ≤ 0.01). Size of circle indicates strength of correlation (the higher the correlation coefficient, the larger the circle). Red and blue indicate the positive and negative correlations, respectively.

According to the correlation analysis results among 20 *L. cubeba* phenotypes (Figure 3), 82 of the 190 pairs of trait combinations had significant correlations, and 59 pairs had extremely significant correlations. There were nine pairs with a correlation coefficient greater than 0.80, indicating an extremely significant positive correlation: LA, leaf width (LW, distance at the widest part of the leaf), and LL; LW and LL; essential oil yield (EOY) and FY; FY/BD; EOY/BD; FY/BD and FY; EOY/BD; and fresh pulp kernel ratio (FPKR) and FFPR. There were also 22 groups with correlation coefficients greater than 0.5, which are extremely significant correlations, including 3 negative correlation groups and 19 positive correlation groups.

The TH was positively correlated with BD (r = 0.41 **), CD (r = 0.51 **), MC (r = 0.32 **), FFPR (r = 0.24 **), FPKR (r = 0.30 **), FY (r = 0.22 **), PL (r = 0.15 *), LP (r = 0.27 **), number of leaf veins(LV, total number of veins on the left and right sides of the midrib from the base of the leaf to the top of the serration) (r = 0.13 *), and EOY (r = 0.19 **) but negatively correlated with TDW (r = −0.24 **) and EOC (r = −0.26 **), indicating that taller *L. cubeba* trees tended to have a larger ground diameter and crown, better fruit characteristics, and high FY and EOY.

There were significant correlations between LA and LL, LW, LP, PL, and LV. The LSI was negatively correlated with LW (r = −0.56 **) and LA (r = −0.38 **), and positively correlated with LV (r = 0.21 **), indicating that the phenotypic characteristics of leaves were closely related.

The TFW was positively correlated with LL (r = 0.14 **), LA (r = 0.17 *), PL (r = 0.20 **), LP (r = 0.15 *), and LV (r = 0.21 *). The TDW was positively correlated with LI (r = 0.13 **) and LP (r = 0.17 *), and the FWC was positively correlated with PL (r = 0.16 *). The EOC was positively correlated with LL (r = 0.13 *). The EOY was positively correlated with LA (r = 0.14 *) and LL (r = 0.16 *), and the EOY/BD was positively correlated with LA (r = 0.17 **), LL (r = 0.21 **), and LW (r = 0.15 *). The FY/BD was positively correlated with LA (r = 0.13 *) and LL (r = 0.17 *). These results indicated that leaf traits affected fruit traits (TFW, TDW, FWC, FY/BD, EOC, EOY, and EOY/BD).

The TFW was positively correlated with FFPR (r = 0.26 **), FPKR (r = 0.21 **), TDW (r = 0.54 **), and FWC (r = 0.38 **). The FFPR was positively correlated with FPKR (r = 0.91 **), and FWC was positively correlated with FFPR (r = 0.75 **), FPKR (r = 0.70 **), and FY (r = 0.15 *). The EOC was positively correlated with TDW (r = 0.28 **) and negatively correlated with TFW (r = −0.24 **), FWC (r = −0.56 **), FFPR (r = −0.46 **), FPKR (r = −0.48 **) and FY (r = −0.15 *). These findings indicated that the phenotypic characteristics of fruit were closely related.

### 2.3. Relationships between Phenotypes and Environmental Factors

#### 2.3.1. Correlations between Phenotypes and Climatic Conditions

We performed correlation analyses to explore the relationships among 20 *L. cubeba* characteristics under different climate conditions (Figure 3). The annual average temperature (AAT, °C) was positively correlated with LL (r = 0.21 **), LP (r = 0.46 **), and PL (r = 0.18 **) and negatively correlated with BD (r = −0.18 **), CD (r = −0.15 *), TFW (r = −0.28 *), and TDW (r = −0.25 **). The annual average maximum temperature (AAMaxT, °C) was positively correlated with LL (r = 0.13 *), LP (r = 0.40 **), and PL (r = 0.18 **) and negatively correlated with CD (r = −0.15 *), TFW (r = −0.18 *), and TDW (r = −0.27 **). The annual average minimum temperature (AAMinT, °C) was positively correlated with TH (r = 0.14 *), LL (r = 0.25 *), LW (r = 0.23 **), LA (r = 0.20 **), PL (r = 0.16 *), and LP (r = 0.46 **) and negatively correlated with BD (r = −0.23 **), TFW (r = −0.28 **), and TDW (r = −0.29 **). The annual average relative humidity (AARH, %) was positively correlated with BD (r = 0.16 *), CD (r = 0.17 **), LW (r = 0.23 **), LA (r = 0.21 *), FFPR (r = 0.20 **), FPKR (r = 0.19 **), and FWC (r = 0.16 *) and negatively correlated with LSI (r = −0.23 **) and EOC (r = −0.15 *). The AAR was positively correlated with LSI (r = 0.21 **) and EOC (r = 0.25 **) and negatively correlated with BD (r = −0.17 **), CD (r = −0.29 **), TH (r = −0.35 **), LW (r = −0.17 *), LA (r = −0.19 **), LP (r = −0.17 *), TFW (r = −0.32 **), FFPR (r = −0.21 **), FPKR (r = −0.22 **), FWC (r = −0.23 **), FY (r = −0.17 *), and EOY (r = −0.15 *). These results showed that climate conditions were closely related to the phenotype of *L. cubeba*. The AAR was significantly negatively correlated with FY and EOY, indicating that the yield was relatively low in areas with sufficient water. The AARH was significantly negatively correlated with EOC, indicating that excessive relative humidity was not conducive to the accumulation of essential oil.

#### 2.3.2. Correlations between Phenotypes and Geographic Features

We also explored the relationship between geographic features and 20 phenotypes of *L. Cubeba* by correlation analysis (Figure 3). We found that north latitude was positively correlated with LL (r = 0.16 *), LW (r = 0.14 *), TDW (r = 0.39 **), EOC (r = 0.51 **), and EOY/BD (r = 0.36 **) and negatively correlated with BD (r = −0.23 **), CD (r = −0.23 **), TH (r = −0.42 **), TFW (r = −0.18 **), FFPR (r = −0.38 **), FPKR (r = −0.43 **), FWC (r = −0.58 **), FY (r = −0.25 **), and FY/BD (r = −0.15 *). East longitude was positively correlated with LSI (r = 0.21 *), TDW (r = 0.20 **), and EOC (r = 0.48 **) and negatively correlated with BD (r = −0.22 **), CD (r = −0.32 **), TH (r = −0.40 **), LW (r = −0.18 **), LA (r = −0.21 **), PL (r = −0.18 **), TFW (r = −0.37 **), FFPR (r = −0.39 **), FPKR (r = −0.45 **), FWC (r = −0.56 **), FY (r = −0.33 **), FY/GD (r = −0.22 **), EOY (r = −0.28 **), and EOY/BD (r = −0.17 *). Elevation was positively correlated with BD (r = 0.26 **), CD (r = 0.25 **), TH (r = 0.34 **), TFW (r = 0.43 **), FFPR (r = 0.24 **), FPKR (r = 0.27 *), FWC (r = 0.46 **), FY (r = 0.23 **), and EOY (r = 0.13 *) and was proportionally negatively correlated with LL (r = −0.20 **), LW (r = −0.15 *), and EOC (r = −0.45 **). These findings showed that, the higher the elevation, the greater the TDW, FY, and EOY. There were no significant correlations between leaf characteristics (LL, LP, and LV) and geographical factors.

#### 2.3.3. Correlations between Phenotypes and Soil Physicochemical Properties

The relationships between soil physicochemical properties and 20 phenotypes of *L. cubeba* were determined by correlation analysis (Figure 3). The soil water content (SWC) was significantly positively correlated with CD (r = 0.22 **), TH (r = 0.26 **), TFW (r = 0.21 **), FFPR (r = 0.26 **), FFPR (r = 0.26 **), and FWC (r = 0.33 **) and negatively correlated with LL (r = −0.23 **), LW (r = −0.14 *), LP (r = −0.34 **), and EOC (r = −0.42 **). The SBD was positively correlated with LL (r = 0.33 **), LW (r = 0.44 **), LP (r = 0.14 *), PL (r = 0.18 **), LA (r = 0.41 **), FWC (r = 0.24 **), FY (r = 0.17 **), FY/BD (r = 0.17 **), EOY (r = 0.24 **), and EOY/BD (r = 0.25 **) and significantly negatively correlated with LSI (r = −0.34 **) and TDW (r = −0.19 **). The soil maximum water holding capacity (SMaxWHC) was positively correlated with CD (r = 0.15 **), TH (r = 0.20 **), LSI (r = 0.20 **), FPKR (r = 0.14 *), FFPR (r = 0.16 *), and FWC (r = 0.14 *) and negatively correlated with LL (r = −0.32 **), LW (r = −0.34 **), LA (r = −0.30 **), LP (r = −0.24 **), EOC (r = −0.36 **), and EOY/BD (r = −0.15 **). The soil minimum water holding capacity (SMinWHC) was positively correlated with CD (r = 0.15 *), TH (r = 0.15 *), TFW (r = 0.16 *), FPKR (r = 0.17 *), FFPR (r = 0.19 **), and FWC (r = 0.16 *) and significantly negatively correlated with LL (r = −0.27 **), LW (r = −0.24 **), LA (r = −0.20 **), LP (r = −0.26 **), and EOC (r = −0.40 **). The SP was positively correlated with BD (r = 0.13 *), CD (r = 0.24 **), TH (r = 0.23 **), TFW (r = 0.15 *), FPKR (r = 0.41 **), FFPR (r = 0.41 **), FWC (r = 0.49 **), FY (r = 0.20 **), and FY/BD (r = 0.24 *) and significantly negatively correlated with LSI (r = −0.13 *), LP (r = −0.18 **), TDW (r = −0.31 **), and EOC (r = −0.51 **). The soil pH (SpH) was positively correlated with LSI (r = 0.28 **), LP (r = 0.26 **), LV (r = 0.17 **), TDW (r = 0.16 *), EOC (r = 0.19 **), EOY (r = 0.20 **), and EOY/BD (r = 0.21 **) and negatively correlated with LW (r = −0.15 *), FFPR (r = −0.15 *), and FWC (r = −0.19 **), and FFPR (r = −0.13 *). The soil total carbon content (STOC) was positively correlated with CD (r = 0.17 *), TH (r = 0.30 *), LSI (r = 0.23 **), FFPR (r = 0.21 **), FPKR (r = 0.22 **), and FWC (r = 0.23 *) and negatively correlated with LL (r = −0.24 **), LW (r = −0.31 **), and EOC (r = −0.39 **). The soil total nitrogen content (STNC) was positively correlated with TH (r = 0.26 **), LSI (r = 0.27 **), LV (r = 0.16 *), FPKR (r = 0.16 *), FFPR (r = 0.15 *), and FWC (r = 0.15 *) and negatively correlated with LL (r = −0.20 **), LW (r = −0.29 **), LA (r = −0.26 **), and EOC (r = −0.25 **). The soil total phosphorus content (STPC) was significantly positively correlated with CD (r = 0.19 **), TH (r = 0.25 **), LSI (r = 0.29 **), LV (r = 0.19 **), FFPR (r = 0.18 **), FPKR (r = 0.23 **), and FWC (r = 0.18 **) and significantly negatively correlated with LL (r = −0.30 **), LW (r = −0.41 **), LA (r = −0.37 **), PL (r = −0.18 **), and EOC (r = −0.19 **). The soil total potassium content (STKC) was positively correlated with LL (r = 0.21 **), LW (r = 0.14 *), LA (r = 0.16 *), FY (r = 0.14 *), and EOY (r = 0.18 **) and negatively correlated with TH (r = −0.15 *), LP (r = −0.17 **), FPKR (r = −0.12 *), and FWC (r = −0.18 **). These results showed that most *L. cubeba* phenotypes were significantly correlated with soil factors, but BD was not significantly correlated with any of the soil physicochemical properties.

### 2.4. Path Analysis to Detect Relationships among Environmental Factors and Major Phenotypes

**Figure 4:**
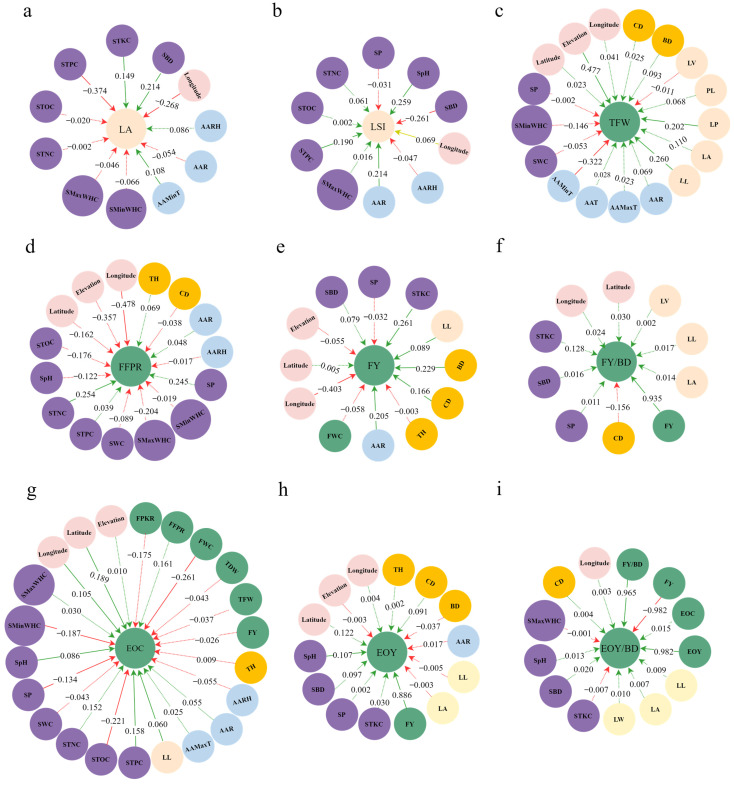
Results of path analysis of phenotypes of *L. cubeba*. Solid arrows represent the direct effects of environmental factors on the phenotype, and dashed arrows represent the correlation effects among the direct factors. The green line indicates a positive effect, and the red line indicates a negative effect. (**a**) LA, (**b**) LSI, (**c**) TFW, (**d**) FFPR, (**e**) FY, (**f**) FY/BD, (**g**) EOC, (**h**) EOY, (**i**) EOY/BD.

In the path analysis, the direct path coefficient was greater than the indirect path coefficient. Some environmental factors were direct influences on major phenotypes, and some were indirect influences (Table S6). Among the phenotypic characters, CD and BD were direct influences on FY (Figure 4e). Among climate factors, AAR directly influenced two phenotypes: LSI, an indicator of leaf shape (Figure 4b), and FY, a fruit phenotype (Figure 4e). AAMinT directly influenced LA and TFW (Figure 4a,c). Among the topographic factors, elevation directly affected TFW (Figure 4c), latitude directly affected EOC (Figure 4g), and longitude directly affected five phenotypes (LA, LSI, FFPR, FY, and EOC) (Figure 4a,b,d,e,g).

Among the soil factors, STKC directly influenced LA and FY (Figure 4a,e), where LA is a measure of leaf size. STNC directly influenced FFPR (Figure 4d), and STPC and SBD directly influenced LA and LSI, respectively (Figure 4a,b), both of which are leaf shape phenotypes. SpH directly influenced EOC and EOY (Figure 4g,h), which are important fruit phenotypes. EOC was also directly influenced by STPC, SpH, SMinWHC, SP, and STOC (Figure 4g); their direct path coefficients on EOC were 0.158, 0.086, −0.187, −0.134, and −0.221, respectively (Table S6).

The leaf phenotypes LL and LP were direct influences on TFW (Figure 4c), and LL was a direct influence on FY and EOC (Figure 4e,g). FY was a direct influence on FY/BD, EOY, and EOY/BD (Figure 4f,h), and FWC was a direct influence on EOC (Figure 4g). EOY and FY/BD directly influenced EOY/BD (Figure 4i). Among the nine fruit phenotypes, FY/BD and EOY/BD were not influenced by climatic factors (Figure 4f,i). FY/BD directly influenced FY (Figure 4f). None of the soil factors directly influenced TFW, FY/BD, or EOY/BD (Figure 4c,f,i).

### 2.5. Environmental Factors Affecting Phenotypic Characteristics

The original data of phenotypic characteristics were subjected to DCA. As summarized in Table S8, the gradient lengths of the sorting axes were all less than 3 (DCA 1 = 1.49, DCA 2 = 1.15, DCA 3 = 0.58, and DCA 4 = 0.61), indicating that the data were suitable for RDA with a linear model. The 20 phenotypic characteristics of *L. cubeba* were used as response variables for the RDA. The explanatory variables consisted of 17 environmental factors, and the soil factor SpH. The results obtained using the RDA model were significant at the *p* = 0.001 level and revealed that most of the environmental factors tested here contributed to the phenotypic diversity of *L. cubeba*. All 18 environmental factors together explained 36.08% of the variation in the phenotypic characteristics of *L. cubeba*. The first axis explained 11.71% of the variation in phenotypic characteristics, and the second axis explained 9.43% (Table S9). Thus, the first two axes in the RDA provided evidence for the relationship between environmental factors and the phenotypic characteristics of *L. cubeba*.

**Figure 5:**
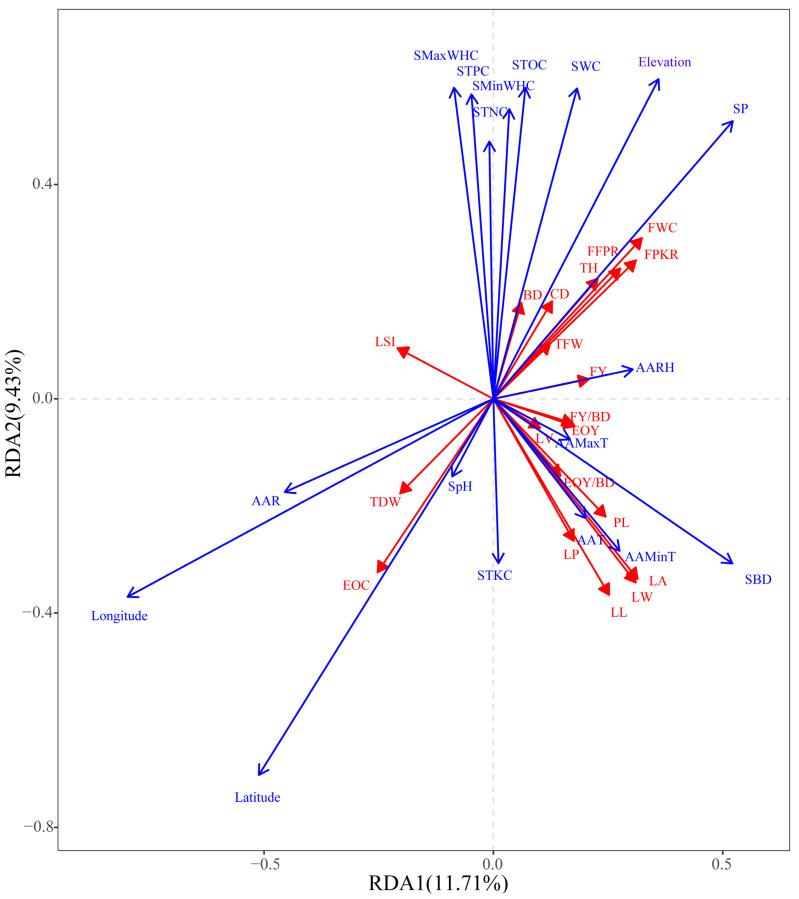
Redundancy analysis to detect relationships between environmental factors and phenotypic characteristics of *L. cubeba*. Data for 18 environmental variables were included in the analysis to detect relationships between environmental factors and phenotypic characteristics of *L. cubeba*. RDA1 explained 11.71% of phenotypic diversity, and RDA2 explained 9.43%.

According to the correlations between environmental factors and phenotypic characteristics of *L. cubeba*, the forward selection method was used to select the most important environmental factors contributing to variations in phenotype among provenances (Figure 5). RDA1 was strongly positively associated with variables such as FWC, FPKR, LA, LW, SP, SBD, and elevation but highly negatively associated with EOC, TDW, LSI, AAR, east longitude, and north latitude. RDA2 was strongly positively associated with elevation, SWC, STOC, STPC, SMaxWHC, and SP but strongly negatively associated with east longitude, north latitude, EOC, LL, and LW. Thus, east longitude, north latitude, elevation, AAR, SBD and SP were identified as the most important environmental factors affecting the phenotypic characteristics of *L. cubeba*.

**Figure 6:**
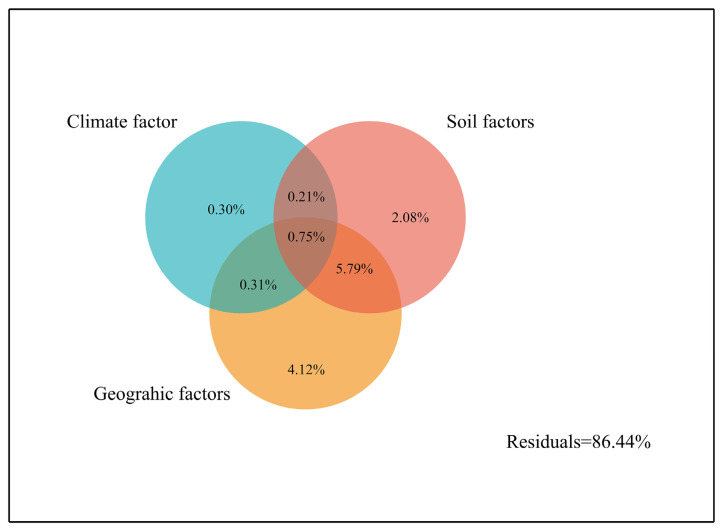
Variance partitioning analysis of six dominant environmental factors. The six dominant environmental factors were selected to variance partitioning analysis. Longitude, latitude, and elevation classified as geographic factors, SP and SBD classified as soil factors, while only AAR belonged to climatic factor.

Based on the results of the RDA analysis, the variance decomposition of important environmental factors revealed that geographic factors explained a higher proportion of variance (4.12%) than did soil factors (2.08%) and climate factors (0.30%) (Figure 6). The combined effects of geographic features and climatic factors explained 0.313% of variation; those of geographic features and soil factors explained 5.79% of variation; and those of climatic factors and soil factors explained 0.96% of variation. As shown in Figure 6, the combined effects of the six dominant environmental factors had a relatively small influence on the variations in phenotypic characteristics of *L. cubeba*.

## 3. Materials and Methods

### 3.1. Plant Materials

Leaf and fruit samples of *L. cubeba*, as well soil samples, were collected from 36 provenances in nine provinces (Anhui, Fujian, Guangxi, Guizhou, Hunan, Jiangxi, Sichuan, Yunnan, and Zhejiang) during 2020 and 2021 (Figure 1). The geographic distribution of sampling sites is shown in Figure 1. The sampling sites spanned the area south of the Huai River in China at latitude 8°52′ and longitude 20°13′ in southern China. The distance between samples was at least 100 m to avoid collecting clones [10,28].

### 3.2. Phenotypic Characteristics

#### 3.2.1. Leaf and Tree Morphological Characteristics

Because of the strong similarity of the leaves, we randomly selected at least three trees from each origin to harvest leaf samples. At least 30 mature leaves from each tree were placed in a sealed bag and filled with silica gel to prevent decay and deterioration. A camera (Canon, Tokyo, Japan) was used to photograph 306 trees from the 36 provenances. The leaves in the images were analyzed using Digizimer v5.7.2 software (https://www.digimizer.com; accessed on 3 October 2021). Seven leaf phenotypic parameters were selected as leaf phenotypic traits in this study. LL, LW, PL, LA, LP, and LV. All these phenotypic parameters were measured directly using Digizimer v5.7.2 software. Based on the LL and LW, the LSI was calculated [29]. We also investigated the growth characteristics of *L. cubeba* by measuring the TH using a tower ruler, the BD using an electronic caliper, and the CD of the tree using a tape measure.

#### 3.2.2. Fruit Characteristics

*L. cubeba* fruits are small and sparsely distributed on the tree, so it is difficult to collect all the fruits from a single tree. In this study, all fruits on a fruit-bearing branch were collected and weighed using an electronic scale. The yield per plant was calculated from the number of fruit-bearing branches and the fruit yield of the fruit-bearing branches collected. The yield per unit ground diameter was obtained by dividing the yield per plant by the ground diameter of the tree. Each essential oil (EO) sample was extracted from approximately 100 g fresh fruit. Three duplicate samples are prepared for each tree. The EO was extracted by steam distillation (feed to liquid ratio = 1:10) for 150 min, and then the EO was separated out. The EOC was calculated as described by Huang et al. (2020) [30]. The fruits were collected from 750 trees in this survey, and the TFW and TDW were calculated from fresh and dry weight values of 1000 de-stemmed *L. cubeba* fruits. The FWC, FPKR, and FFPR were calculated from 50 de-stemmed fruits and measured for three replicates of fruit from each tree (Table S2).

### 3.3. Environmental Factors

Information on the geographical features for each site was collected using 2bulu software (https://www.2bulu.com; accessed on 1 July 2020). Data on climatic variables were obtained from the China Meteorological Administration (http://www.cma.gov.cn; accessed on 2 December 2021), which provided the closest approximation for the sites based on data collected over a wide geographical area. The climatic variables obtained for each collection site were AAT, AAMaxT, AAMinT, AARH, and AAR. The geographical characteristics and climatic conditions of each provenance collection site are summarized in Table S1.

In this study, inter-rhizosphere soil samples were randomly collected from 34 of the 36 provenance collection sites (all except for GZ-TJ and HN-DX). Soil physical properties were measured using the ring knife method (ring knife volume of 100 cm) and the maceration method. SWC, SBD, SMaxWHC, SMinWHC, and SP were calculated using the formulae described by Reichert et al. (2023) [31]. SpH was measured using a pH meter. STOC was determined as described by Visconti et al. (2022) [32]. Soil samples were digested with H_2_SO_4_-HClO_4_ and STPC, STNC, and STKC were determined according to the methods of Wang et al. (2023) [33] and Sukkaew et al. (2022) [34].

### 3.4. Statistical Analysis

Descriptive analyses were used to evaluate 20 phenotypes for all samples. We used R software (https://www.r-project.org; accessed on 3 November 2021) to calculate the mean, standard error (SE), standard deviation (SD), extreme deviation, the coefficient of variation (CV), and Shannon–Weiner diversity index (*H′*) of the measured data. The CV indicates the degree of dispersion of phenotypic characteristics as described by Schillaci et al. (2022) [35]. The H′ value reflects the diversity of phenotypic traits in each plant population as described by Hamil et al. (2021) [36] and Maru et al. (2022) [37]. We performed nested analysis of variance (ANOVA), multiple comparison analysis (LSD test, *p* ≤ 0.05), and cluster analysis (hierarchical clustering, Euclidean squared distance) on all data for the 20 main phenotypes of *L. cubeba*. We performed multiple regression pass analysis, detrended correspondence analysis (DCA), redundancy analysis (RDA), and variance decomposition analysis (VPA) on the data for 18 environmental factors and 20 major phenotypes of *L. cubeba* using tools in the R.4.20 statistical environment. Pearson’s correlation analysis was performed using OriginPro Learning Edition 2022 software (https://www.originlab.com; accessed on 3 April 2022).

## 4. Discussion

### 4.1. Phenotypic Diversity of L. cubeba Characteristics

Plant phenotypic characteristics are controlled by both genetic and environmental factors. The phenotype directly reflects the genetic diversity of plants, which is important for screening excellent germplasm resources [38,39]. This is the first comprehensive and systematic study of 20 traits of *L. cubeba*, including its growth, fruit, and leaf characteristics. The dataset generated in this study allowed us to accurately assess the phenotypic diversity of *L. cubeba* germplasm resources distributed in nine provinces in China.

The H′ and CV of the phenotypic characteristics of *L. cubeba* were calculated, and revealed wide phenotypic diversity among the 36 provenances of *L. cubeba*. The average H′ of the 20 phenotypes was 1.82. It was lower than the H′ values reported for *L. caerulea* [40] (H > 2.00) and *C. pinnatifida* [41] (H > 4.49) and higher than that reported for E. japonica [42]. This indicates that *L. cubeba* still shows rich diversity, despite germplasm losses. However, the H′ value is closely related to the sample size, highlighting that an appropriate sample size should be selected in germplasm surveys [43]. The CV of 20 phenotypes of *L. cubeba* ranged from 7.15% to 218.84%, and there was wide phenotypic variation among the natural germplasm, especially in the seven phenotypic traits that are greatly influenced by age (e.g., ground diameter, crown width, tree height, and fruit yield). After the exclusion of those seven traits, the CV of the remaining 13 phenotypes ranged from 7.15% to 53.18%, similar to the values reported for *X. sorbifolium* [44] (12.80–3.25%). This suggests that *L. cubeba* has rich phenotypic variation under different environments and indicates that it is highly adaptable in complex habitats [45].

The mean values of the H′ value for the 20 phenotypes across all of the provenances ranged from 1.35 (EOY/BD) to 1.85 (FFPR), and the mean CV ranged from 5.34% (FFPR) to 110.80% (FY). These findings revealed that *L. cubeba* is susceptible to population differentiation. We confirmed through field surveys that *L. cubeba* populations have increased resistance to gene flow within populations due to the effects of destructive harvesting. This has led to population differentiation and phenotypic trait variation.

Based on the results of the nested ANOVA, we found that the phenotypic diversity of *L. cubeba* was mainly derived from interspecific variation (*p* < 0.01), where growth characteristics, fruit characteristics (FY, FY/BD, and EOY/BD), and leaf characteristics (LP, PL, and LSI) were jointly influenced by intra- and interspecific variation. Previous studies have shown that *Pyrus spinosa* [46] exhibits great phenotypic variation across habitats. We hypothesize that the abundant variation in our surveyed *L. cubeba* samples is closely related to the environmental conditions at their growing sites. Further studies are required to reveal the intrinsic link between the phenotypic diversity of *L. cubeba* and the environmental conditions in its habitat. In addition, extensive gene exchange within dioecious plant populations can lead to a high degree of genetic heterozygosity, resulting in phenotypic diversity within populations [47,48].

### 4.2. Environmental Factors Affecting Fruit and Leaf Characteristics

Species differ in their sensitivity and adaptability to different environments, and phenotypic diversity reflects the adaptability of populations to different environmental conditions. Therefore, phenotypic diversity can be used to some extent as an indicator of species’ adaptability to complex environments [49,50]. In this study, analyses of the overall age composition of *L. cubeba* population revealed a predominance of younger trees. However, *L. cubeba* in the western part of the survey area were less affected by anthropogenic activities and were mostly in their prime, with larger trees and higher fruit yield. In another study, geographical location and environmental selection pressure were identified as key factors leading to phenotypic differentiation among *L. cubeba* populations [51]. Those results are consistent with the results of the Pearson’s correlation analysis in this study, indicating a close relationship between geographical location and *L. cubeba* phenotype. Previous studies have detected significant correlations between geographic location and climatic factors and have shown that climatic factors affect secondary metabolites [52,53]. Soil provides nutrients and the matrix for plants to grow and develop. Other studies have shown that the physicochemical properties of soil affect plant variability [54,55,56]. In the present study, we found that most *L. cubeba* phenotypes were significantly correlated with the geographic features of the sampling sites.

The results of the pathway analysis showed that STNC, STKC, STPC, and STOC were important direct influences on the phenotype of *L. cubeba*, while SBD, SP and SpH were independent indirect influences. The 18 environmental factors evaluated in this study collectively explained nearly one-third of the phenotypic variation in *L. cubeba*, with eastern longitude, northern latitude, elevation, AAR, SBD, and SP being the most important factors. Thus, these environmental factors may be important contributors to the phenotypic diversity of *L. cubeba*. The *L. cubeba* trees with higher EOC were often distributed at higher latitudes and lower elevations, whereas those with larger leaves tended to grow at lower latitudes. Our results show that geographic features have a direct and strong influence on the phenotypic characteristics of *L. cubeba*. In addition, the climate varies among geographical locations, and thus indirectly influences the phenotypes of *L. cubeba*. Previous studies have also linked environmental factors to fruit phenotypes and metabolites, particularly terpenoids [10,57,58]. For example, AAR was found to increase with latitude and longitude, and AAR affected the contents of volatile components and terpene compounds. Other studies also found that lower AAR was related to higher EOC [59]. In this study, we found that SBD increased with decreasing latitude, and SBD significantly affected leaf phenotypes. We found that SP was negatively correlated with latitude and longitude, but positively correlated with elevation and negatively correlated with EOC. Previous studies have shown that soil porosity has a significant effect on the rooting environment and that root growth has a significant effect on the variation in phenotypes [59,60].

Other environmental factors not evaluated in this study, such as plant rhizosphere microorganisms, light factors, and the presence of other plants’ roots in the rhizosphere, may also affect the phenotypic characteristics of *L. cubeba* [61,62,63]. In addition, genotypic differences may be the most direct factor influencing variation in phenotypic traits. Previous studies on wheat have shown that genotype significantly affects phenotype [64]. In conclusion, our results show that six habitat factors (longitude, latitude, elevation, AAR, SBD, and SP) strongly influence *L. cubeba* phenotypic traits. This information will be useful for selecting excellent germplasm resources and for optimizing the cultivation conditions for *L. cubeba*.

Fruit are the sinks for photosynthetic products that accumulate in plant leaves [65]. Our study showed that trees with longer leaves had better fruiting characteristics (higher TFW, FY, and EOC). In future studies, it will be important to explore how different environmental factors affect phenotypic traits of *L. cubeba* trees of the same genotype (e.g., using a population of asexually propagated seedlings) and to determine differences in the phenotypes of genotypically different *L. cubeba* lines growing in the same environment. The results of such studies will be useful for devising strategies to conserve genetic diversity, to select resources for advanced breeding techniques, and to improve the supply of this economically important species [66,67].

## 5. Conclusions

*L. cubeba* is an important woody spice plant resource in China, and displays wide phenotypic diversity in terms of tree shape, leaves, and fruit. Among these, the fruit is the most important component because it is the source of valuable essential oil. Our results demonstrated that environmental factors significantly affect phenotypic traits. Leaf and fruit characters had a more significant geographical distribution, with larger LA and larger TFW in provenances in southwest China but higher EOC in provenances in southeast China. Among the 36 provenances, SC-KJ, SC-HJ, and SC-LS had larger LA, YN-SM and YN-XC had larger TFW, and HN-DX had the highest EOC.

In this study, STPC, SpH, SBD, AAR, and latitude were identified as the important factors directly generating LSI diversity, whereas latitude and STNC were identified as the important factors generating FFPR diversity. The most important factors generating EOC diversity were SP, SMinWHC, SpH, STOC, STPC, LL, FWC, latitude, and longitude. It will be important to explore why and how these environmental features are direct drivers of the phenotypic diversity observed in this work, as stated above. The redundancy analysis showed that longitude, latitude, elevation, AAR, SBD, and SP are the important factors affecting the phenotype of *L. cubeba*. Further research should explore the mechanisms of phenotype formation in *L. cubeba*. Such information will provide a theoretical basis for breeding high-quality *L. cubeba* cultivars and for developing optimal conditions for their cultivation.

## Data Availability

All generated data are included in the supplementary data to this article.

## Supplementary Materials

The following supporting information can be downloaded at: https://www.mdpi.com/article/10.3390/plants12213781/s1, Table S1. Geographical locations and climatic conditions of provenances in China; Table S2. Variation analysis on phenotypic traits of *L. cubeba*; Table S3. CV of phenotypic traits within provenances of *L. cubeba*; Table S4. Shannon–Wiener index (*H′*) of phenotypic traits within provenances of *L. cubeba*; Table S5. Nested variance analysis for phenotypic traits of *L. cubeba*; Table S6. Multiple comparison on leaf and growth characteristics between provenances of *L. cubeba*; Table S7. Multiple comparison on fruit characteristics between provenances of *L. cubeba*; Table S8. The result of path analysis for LA of *L. cubeba*; Table S9. The result of path analysis for LSI of *L. cubeba*; Table S10. The result of path analysis for TFW of *L. cubeba*; Table S11. The result of path analysis for FFPR of *L. cubeba*; Table S12. The result of path analysis for FY of *L. cubeba*; Table S13. The result of path analysis for FY/BD of *L. cubeba*; Table S14. The result of path analysis for EOC of *L. cubeba*; Table S15. The result of path analysis for EOY of *L. cubeba*; Table S16. The result of path analysis for EOY/BD of *L. cubeba*; Table S17. Results on detrended correspondence analysis of environmental factors; Table S18. Results on detrended correspondence analysis of environmental factors.

## Abbreviations

BD, Basal diameter; CD, crown diameter; TH, tree height; EOC, essential oil content; EOY/BD, essential oil yield/basal diameter; EOY, essential oil yield; FPKR, fresh pulp kernel ratio; FFPR, fresh fruit pulp rate; FWC, water content of fruit; FY, fruit yield; FY/BD, fruit yield/basal diameter; TFW, thousand-grain fresh weight; TDW, thousand-grain dry weight; LA, leaf area; LL, leaf length; LW, leaf width; LP, leaf perimeter; PL, petiole length; LSI, leaf shape index; LV, number of leaf veins; AAT, annual average temperature; AAMaxT, annual average maximum temperature; AAMinT, annual average minimum temperature; AAR, annual average rainfall; AARH, annual average relative humidity; SWC, soil water content; SBD, soil bulk density; SP, soil porosity; SMaxWHC, soil maximum water-holding capacity; SMinWHC, soil minimum water-holding capacity; STOC, soil total carbon content; SpH, soil pH; STNC, soil total nitrogen content; STPC, soil total phosphorus content; STKC, soil total potassium content.
