# Supplementary material for: Main Habitat Factors Driving the Phenotypic Diversity of Litsea cubeba in China
Source: Plants (Basel). 2023 Nov 6;12(21):3781. doi: 10.3390/plants12213781 (PMC10648399; doi:10.3390/plants12213781)
Supplement: Supplementary file 1 [file plants-12-03781-s001.zip › plants-2663986-supplementary.pdf]

Supplementary Materials: Main habitat factors driving the phenotypic diversity of *Litsea cubeba* in China

NO.	Supplementary materials name
Table S1	Geographical locations and climatic conditions of provenances in China
Table S2	Variation analysis on phenotypic traits of *L. cubeba*
Table S3	CV of phenotypic traits within provenances of *L. cubeba*
Table S4	Shannon–Wiener index (*H′*) of phenotypic traits within provenances of *L. cubeba*
Table S5	Nested variance analysis for phenotypic traits of *L. cubeba*
Table S6	Multiple comparison on leaf and growth characteristics between provenances of *L. cubeba*
Table S7	Multiple comparison on fruit characteristics between provenances of *L. cubeba*
Table S8	The result of path analysis for LA of *L. cubeba*
Table S9	The result of path analysis for LSI of *L. cubeba*
Table S10	The result of path analysis for TFW of *L. cubeba*
Table S11	The result of path analysis for FFPR of *L. cubeba*
Table S12	The result of path analysis for FY of *L. cubeba*
Table S13	The result of path analysis for FY/BD of *L. cubeba*
Table S14	The result of path analysis for EOC of *L. cubeba*
Table S15	The result of path analysis for EOY of *L. cubeba*
Table S16.	The result of path analysis for EOY/BD of *L. cubeba*
Table S17.	Results on detrended correspondence analysis of environmental factors
Table S18	Results on detrended correspondence analysis of environmental factors

Table S1. Geographical locations and climatic conditions of provenances in China

Provenance	County acronym	Sample size	Latitude (°N)	Longitude (°E)	Elevation (m)	AAT (℃)	AAMaxT (℃)	AAMinT (℃)	AARH (%)	AAR (mm)
Dongzhi County, Anhui	AH-DZ	18	30°11′19.72″	117°14′19.64″	313.8	17.1	21.2	14.0	76.0	1485.2
Jixi County, Anhui	AH-JX	25	29°58′24.17″	118°31′56.21″	169.5	16.1	22.1	11.9	82.0	1797.6
Qimen County, Anhui	AH-QM	21	29°51′25.85″	117°47′07.51″	221.4	16.6	20.9	13.3	77.0	1392.5
Qingyang County, Anhui	AH-QY	10	30°40′35.51″	117°49′05.23″	57.9	16.5	21.7	12.5	78.0	1747.9
Xiuning County, Anhui	AH-XN	25	29°50′46.75″	118°10′34.03″	402.3	16.6	22.0	12.6	76.0	1652.4
Changting County, Fujian	FJ-CT	17	25°01′04.15″	116°19′41.12″	528.8	18.5	24.2	14.6	80.0	1712.1
Jianou County, Fujian	FJ-JO	20	27°02′44.02″	118°07′53.36″	277.5	19.1	24.8	15.2	81.0	1673.6
Liancheng County, Fujian	FJ-LC	25	25°24′14.94″	116°10′57.22″	481.0	20.3	25.9	16.7	76.0	1666.9
Shaxian County, Fujian	FJ-SX	24	26°27′37.69″	117°43′50.05″	208.1	19.6	25.5	15.7	81.0	1692.2
Zhenghe County, Fujian	FJ-ZH	17	27°22′47.28″	118°38′56.69″	545.0	18.8	24.6	14.8	77.0	1628.2
Guangyang County, Guangxi	GX-GY	22	25°29′37.54″	111°13′46.74″	590.8	18.0	22.7	14.7	79.0	1542.9
Dafang County, Guizhou	GZ-BL	22	27°18′05.51″	105°52′14.52″	1949.9	12.1	16.1	9.3	84.0	1085.4
Taijiang County, Guizhou	GZ-TJ	25	26°28′23.63″	108°19′40.94″	747.2	15.9	21.2	12.3	81.0	1096.7
zhenfeng County, Guizhou	GZ-ZF	22	25°21′16.06″	105°32′20.54″	1522.7	16.6	20.6	13.7	79.0	1318.1
zhijin County, Guizhou	GZ-ZJ	25	26°35′57.48″	105°42′38.56″	1632.0	14.3	18.6	11.3	81.0	1355.4
ziyun County, Guizhou	GZ-ZY	25	25°54′52.16″	106°07′22.73″	1224.2	15.5	19.9	12.4	80.0	1250.1
Daoxian County, Hunan	HN-DX	25	25°47′36.31″	111°30′14.65″	706.1	18.8	23.1	15.6	78.0	1503.5
Dexing County, Jiangxi	JX-DX	6	28°49′36.62″	117°25′22.22″	81.0	17.7	23.2	13.7	81.0	1955.5
Wannian County, Jiangxi	JX-WN	25	28°43′42.89″	116°58′04.55″	81.6	17.8	23.0	14.1	81.0	1862.9
Wuyuan County, Jiangxi	JX-WY	14	29°19′17.33″	118°01′01.52″	265.1	17.1	23.1	13.0	82.0	1946.9
Wanzai County, Jiangxi	JX-WZ	19	28°12′38.34″	114°20′42.47″	169.1	17.5	22.6	14.0	82.0	1722.7
Yuanzhou County, Jiangxi	JX-YZ	26	27°57′21.82″	114°13′30.25″	271.1	17.5	22.2	14.0	80.0	1631.4
Hejiang County, Sichuan	SC-HJ	24	28°53′14.14″	106°13′18.30″	950.4	18.0	21.8	15.3	84.0	1122.2
Kaijiang County, Sichuan	SC-KJ	25	30°52′49.87″	107°53′57.84″	476.8	16.7	21.0	13.5	80.0	1238.0
Lingshui County, Sichuan	SC-LS	24	30°07′24.82″	107°04′06.38″	493.0	17.0	21.1	14.1	84.0	1215.0
Lancang County, Yunnan	YN-LC	9	22°25′36.52″	99°48′42.41″	1552.8	19.7	27.5	14.8	77.0	1586.5
Maguang County, Yunnan	YN-MG	23	23°01′40.08″	104°27′37.55″	1408.0	17.2	22.2	14.1	83.0	1286.3
Menghai County, Yunnan	YN-MH	25	22°02′24.25″	100°50′19.07″	1190.8	18.9	26.2	14.0	79.0	1318.5
Mojiang County, Yunnan	YN-MJ	25	22°58′23.30″	101°49′06.78″	1853.7	18.4	24.9	14.0	77.0	1306.0
Simao County, Yunnan	YN-SM	15	22°37′03.29″	100°50′44.23″	1677.2	16.3	21.0	13.1	83.0	1270.6
Xichou County, Yunnan	YN-XC	17	23°33′13.97″	104°44′29.29″	1707.5	18.9	25.2	14.8	77.0	1470.5
Jiangshan County, Zhejiang	ZJ-JS	20	28°17′25.26″	118°34′17.26″	851.6	17.4	22.1	13.9	79.0	1753.8
Liandu County, Zhejiang	ZJ-LD	23	28°27′22.64″	119°59′40.16″	501.7	18.4	23.8	14.5	75.0	1406.0
Longyou County, Zhejiang	ZJ-LY	16	29°13′14.88″	119°04′32.34″	701.9	17.4	22.2	13.7	77.0	1633.0
Qingyuan County, Zhejiang	ZJ-QY	22	27°44′35.05″	119°10′28.31″	1140.6	18.0	23.7	13.8	77.0	1746.9
Suichuan County, Zhejiang	ZJ-SC	24	28°44′28.21″	119°23′31.67″	992.9	17.1	22.9	13.0	78.0	1579.1

AAT, Annual average temperature; AAMaxT, Annual average maximum temperature; AAMinT, annual average minimum temperature; AARH, annual average relative humidity; AAR, annual average rainfall;

Table S2. Variation analysis on phenotypic traits of *L. cubeba*

Traits	Mean ± SE	SD	Range	CV (%)	Shannon–Wiener index (*H'*)
BD (mm)	64.35±1.48	40.53	380.23	62.98	1.85
CD (m^2)	7.43±0.25	6.86	75.70	92.33	1.70
TH (m)	5.1±0.08	2.10	15.10	41.18	2.01
EOC (%)	3.07±0.05	0.84	5.08	27.36	2.07
EOY (g)	74.33±7.54	118.98	896.73	160.07	1.29
EOY/BD (g. cm^{-1})	9.72±0.95	14.92	126.57	153.50	1.33
FPKR	3.83±0.05	1.35	9.60	35.25	1.91
FFPR (%)	77.86±0.2	5.57	43.80	7.15	2.06
FY (kg)	1.38±0.11	3.02	44.72	218.84	0.79
FY/BD (g.cm^{-1})	0.2±0.01	0.36	5.43	180.00	1.04
LA (cm^2)	3.38±0.03	0.58	5.15	17.16	1.88
LL (cm)	8.25±0.11	1.95	13.32	23.64	2.06
LP (cm)	21.95±0.18	3.09	18.00	14.08	1.97
PL (cm)	2.52±0.04	0.77	4.62	30.56	2.05
LSI	1.07±0.02	0.28	1.73	26.17	1.97
LV	51.12±0.48	13.08	97.22	25.59	2.00
LW (cm)	131.57±1.1	30.11	190.62	22.89	1.95
TDW (g)	14.78±0.45	7.86	54.65	53.18	2.03
TFW (g)	18.55±0.41	7.14	37.42	38.49	2.03
FWC (%)	60.34±0.34	9.22	71.20	15.28	2.05

BD, Basal diameter; CD, Crown diameter; TH, Diameter height; EOC, Essential oil content; EOY, Essential oil yield; EOY/BD, Essential oil yield/ Basal diameter; FPKR, Fresh pulp kernel ratio; FFPR, Fresh fruit pulp rate; FWC, Water content of fruit; FY, fresh fruit yield; FY/BD, Fruit yield/ Basal diameter; TFW, Thousand-grain fresh weight; TDW, Thousand-grain dry weight; LA, Leaf area; LL, Leaf length; LW, Leaf width; LP, Leaf perimeter; PL, Petiole length; LSI, Leaf shape index; LV, Number of leaf veins; Mean, mean value; SE, standard error; SD, standard deviation (δ); CV, coefficients of variation, CV＝SD /Mean×100%; Shannon─Wiener index (*H'*) was calculated by the method as described by Hamil et al. (2021).

Table S3. CV (%) of phenotypic traits within provenances of *L. cubeba*

Provenance	BD	CD	TH	EOC	EOY	EOY/BD	FPKR	FFPR	FY	FY/BD	LA	LL	LP	PL	LSI	LV	LW	TDW	TFW	FWC
AH-DZ	69.62	59.65	33.03	25.26	49.85	85.78	20.53	4.85	84.05	110.03	22.00	10.87	14.17	14.86	2.55	15.08	19.95	19.53	20.71	16.04
AH-JX	50.53	87.20	16.95	22.45	30.74	58.74	36.51	10.91	76.94	69.35	16.21	6.66	4.83	7.33	14.85	8.58	12.71	28.20	29.28	19.37
AH-QM	83.41	60.10	22.64	13.02	44.18	70.00	26.98	4.95	65.80	90.97	16.49	9.65	12.70	11.07	9.66	11.95	19.89	17.85	19.88	9.66
AH-QY	46.94	64.15	23.65	15.17	58.88	79.61	31.18	5.33	93.68	85.32	7.88	4.00	4.96	5.85	11.28	2.08	25.90	12.89	13.15	8.70
AH-XN	73.01	69.06	31.72	23.35	33.45	49.21	38.90	6.04	124.89	67.68	26.78	12.52	5.10	14.89	6.80	13.43	19.33	18.28	17.38	11.21
FJ-CT	46.70	66.25	17.58	23.16	60.45	52.32	22.10	5.15	68.83	43.58	41.53	14.51	11.60	19.45	6.95	23.93	20.61	15.77	12.91	8.38
FJ-JO	41.29	59.75	23.75	9.08	34.02	21.74	26.34	6.50	73.25	50.09	31.99	17.53	6.12	18.08	15.03	16.62	19.23	14.34	17.16	6.61
FJ-LC	37.68	63.89	26.13	4.25	24.34	33.17	24.65	5.76	100.38	79.87	29.81	18.80	8.38	19.95	14.67	17.24	24.83	20.26	15.15	10.88
FJ-SX	42.24	61.26	25.50	23.02	72.95	74.60	22.92	4.69	84.99	80.85	16.43	10.97	8.77	8.06	6.35	10.65	19.06	17.64	12.58	10.89
FJ-ZH	59.28	132.76	26.69	—	—	—	34.34	6.56	187.59	112.51	25.59	12.25	16.20	11.31	9.30	17.65	16.64	18.97	16.60	10.44
GX-GY	46.18	71.53	22.95	14.52	71.14	65.92	21.89	5.29	113.07	90.32	41.60	17.32	19.84	18.94	7.32	27.37	18.23	16.76	16.62	10.41
GZ-BL	69.56	92.82	32.34	12.96	100.46	83.18	16.44	3.16	158.16	92.58	14.47	9.44	12.19	10.85	7.11	7.24	13.51	13.52	18.73	6.16
GZ-TJ	39.33	71.39	19.77	22.55	66.65	63.25	16.98	4.22	107.29	96.77	21.32	9.71	11.02	10.12	10.22	14.35	16.81	19.92	19.60	8.08
GZ-ZF	54.17	111.90	67.09	10.71	146.08	81.05	20.87	4.44	145.53	96.84	39.01	18.96	13.97	19.30	8.25	22.42	19.97	20.89	15.72	11.28
GZ-ZJ	51.24	69.10	37.30	29.60	158.98	103.28	20.63	3.98	131.25	105.77	69.85	28.37	17.51	27.74	15.09	41.98	35.44	14.68	19.03	6.10
GZ-ZY	58.17	125.30	47.27	20.87	95.06	94.12	23.29	5.41	139.38	122.55	34.98	15.41	10.69	15.86	11.99	21.16	19.97	13.65	13.99	5.81
HN-DX	42.95	55.64	23.05	15.51	34.57	51.44	30.30	6.29	50.45	47.77	22.49	11.76	5.13	12.23	9.14	10.98	11.91	26.11	20.84	14.15
JX-DX	58.65	100.49	37.90	6.69	28.70	86.53	16.60	3.52	71.77	102.29	19.02	7.39	16.95	8.09	12.31	14.44	2.14	19.29	16.92	2.50
JX-WN	45.95	56.55	32.09	20.23	97.86	55.46	26.36	5.89	105.45	69.01	27.55	11.58	13.81	13.13	12.77	15.40	14.97	19.24	24.77	11.06
JX-WY	31.74	41.49	20.23	3.27	98.89	75.70	28.54	4.98	122.02	104.61	20.42	8.01	26.00	7.97	8.33	20.46	14.42	16.94	13.30	7.16
JX-WZ	26.60	53.81	37.04	27.40	62.32	51.79	33.54	4.57	113.63	89.89	26.95	10.73	24.19	11.95	17.46	20.84	15.93	24.05	19.49	6.29
JX-YZ	43.57	102.41	27.14	22.09	50.09	38.97	14.60	3.30	93.20	72.82	28.22	15.32	11.64	15.54	12.20	17.54	21.08	16.24	14.56	6.98
SC-HJ	20.36	131.84	24.07	12.36	54.60	51.87	23.18	4.80	62.85	56.25	38.96	19.06	13.52	19.16	11.94	23.39	11.37	11.39	13.09	5.51
SC-KJ	36.75	59.72	23.14	18.08	121.21	102.77	27.01	9.16	177.30	146.50	20.62	11.36	8.88	12.02	10.52	9.67	19.75	18.76	27.06	26.77
SC-LS	38.19	57.35	33.05	22.08	101.54	91.97	27.06	5.83	115.82	88.18	34.39	15.53	16.56	14.92	13.65	22.83	26.65	19.85	20.04	11.05
YN-LC	17.29	32.22	23.89	31.02	184.29	179.31	18.84	2.65	230.06	226.45	26.79	16.67	15.43	21.37	19.43	10.95	26.80	32.79	14.25	7.46
YN-MG	39.90	53.09	30.20	39.94	131.67	114.89	23.97	4.69	158.56	141.99	65.05	22.81	9.19	28.08	11.16	29.18	24.57	18.47	16.21	8.31
YN-MH	36.30	43.42	42.61	17.84	69.34	66.95	22.31	3.81	96.72	85.42	19.15	9.76	5.24	10.53	11.33	10.90	14.87	29.73	19.20	6.81
YN-MJ	43.17	83.84	24.22	12.11	42.77	27.98	25.25	5.89	71.90	54.55	38.71	21.78	20.44	24.22	6.65	23.32	15.06	15.49	14.04	2.51
YN-SM	57.96	58.31	36.98	12.61	85.29	101.94	31.54	5.55	137.77	131.84	40.27	23.90	10.23	24.74	5.72	20.00	17.99	24.79	10.71	7.08
YN-XC	31.98	49.95	25.83	17.12	178.51	167.44	25.05	5.72	286.46	268.64	21.20	9.82	11.88	9.14	9.98	15.65	18.68	19.46	17.22	5.16
ZJ-JS	53.36	115.38	37.16	—	—	—	26.06	5.47	52.34	85.88	31.78	39.55	19.82	11.76	13.79	23.10	10.49	19.39	15.70	13.73
ZJ-LD	49.97	65.35	30.14	21.06	29.11	46.56	25.44	9.11	75.03	61.64	19.83	13.69	10.68	14.79	10.06	8.73	19.12	30.12	32.67	6.24
ZJ-LY	66.41	94.01	23.50	18.72	57.19	59.15	12.32	3.46	81.04	70.10	37.90	19.20	10.65	19.45	11.02	18.63	16.62	14.24	16.31	9.03
ZJ-QY	30.10	36.05	23.76	16.12	16.19	31.76	17.43	4.02	55.88	55.08	25.32	20.27	28.28	19.65	14.33	18.36	12.94	15.71	14.96	6.40
ZJ-SC	68.07	74.78	34.30	12.82	38.54	84.02	28.03	6.36	75.32	84.11	25.38	13.79	11.32	14.76	11.29	14.14	18.37	20.58	14.86	12.98
Mean	47.46	73.11	29.57	18.15	74.41	73.60	24.67	5.34	110.80	95.50	29.05	14.97	13.00	15.20	10.85	17.23	18.22	19.33	17.63	9.37

BD, Basal diameter; CD, Crown diameter; TH, Diameter height; EOC, Essential oil content; EOY, Essential oil yield; EOY/BD, Essential oil yield/ Basal diameter; FPKR, Fresh pulp kernel ratio; FFPR, Fresh fruit pulp rate; FWC, Water content of fruit; FY, fresh fruit yield; FY/BD, Fruit yield/ Basal diameter; TFW, Thousand-grain fresh weight; TDW, Thousand-grain dry weight; LA, Leaf area; LL, Leaf length; LW, Leaf width; LP, Leaf perimeter; PL, Petiole length; LSI, Leaf shape index; LV, Number of leaf veins; CV, coefficients of variation, CV＝SD /Mean×100%.

Table S4. Shannon–Wiener index (*H′*) of phenotypic traits within provenances of *L. cubeba*

Provenance	BD	CD	TH	EOC	EOY	EOY/BD	FPKR	FFPR	FY	FY/BD	LA	LL	LP	PL	LSI	LV	LW	TDW	TFW	FWC
AH-DZ	1.83	1.83	1.74	1.73	1.49	1.21	1.98	1.98	1.27	1.46	1.72	1.42	1.49	1.36	1.49	1.10	1.49	1.67	1.99	1.64
AH-JX	1.83	1.70	1.84	1.70	1.83	1.47	1.79	1.85	1.84	1.90	1.43	1.47	1.61	1.61	1.61	1.30	1.61	1.61	1.72	1.93
AH-QM	1.43	1.77	1.88	1.56	1.61	1.89	1.84	2.02	1.78	1.55	1.84	1.70	1.42	1.83	1.56	1.70	1.70	1.64	1.82	1.86
AH-QY	1.75	1.70	1.50	1.04	1.04	1.04	1.28	1.50	1.36	1.22	1.70	1.04	1.39	0.69	1.04	1.04	1.39	1.04	1.83	1.47
AH-XN	1.42	1.78	1.83	1.39	1.39	1.39	1.62	1.97	1.40	1.43	1.92	1.83	1.75	1.61	1.42	1.54	1.61	1.56	1.91	1.71
FJ-CT	1.59	1.81	1.79	1.10	1.10	1.10	1.78	1.81	1.68	2.01	1.82	1.79	1.56	1.33	1.01	1.10	1.33	1.24	1.96	1.96
FJ-JO	1.70	1.85	1.87	1.61	1.61	1.33	2.04	1.69	1.85	1.99	2.00	1.74	1.74	1.74	1.68	1.74	1.68	1.89	1.87	1.85
FJ-LC	1.70	1.87	1.85	1.10	0.64	1.10	1.94	1.87	1.51	1.70	1.93	1.50	1.61	1.36	1.36	1.49	1.61	1.63	1.84	1.87
FJ-SX	1.59	1.93	1.70	1.50	1.28	1.22	1.91	1.99	1.71	1.64	2.02	1.70	1.64	1.61	1.70	1.98	1.56	1.70	1.72	1.79
FJ-ZH	1.25	1.40	1.96	—	—	—	1.56	1.84	0.79	1.48	1.87	1.89	1.70	1.70	1.50	1.70	1.50	1.70	1.72	1.99
GX-GY	1.95	1.44	1.51	1.55	1.55	1.48	1.78	1.71	1.36	1.73	1.86	1.55	1.35	1.35	1.08	1.28	1.55	1.55	1.92	1.80
GZ-BL	1.59	1.57	1.89	1.55	1.28	1.28	1.87	1.96	1.16	1.49	1.84	1.28	1.55	1.48	1.48	1.28	1.48	1.31	1.90	1.83
GZ-TJ	1.83	1.90	2.00	1.64	1.47	1.50	1.92	1.94	1.52	1.48	1.83	1.50	1.64	1.56	1.70	1.70	1.47	1.70	1.70	1.86
GZ-ZF	1.75	1.45	1.31	1.48	1.28	1.75	1.85	1.90	1.40	1.43	2.01	1.55	1.75	1.55	1.35	1.50	1.75	1.55	1.97	1.74
GZ-ZJ	1.84	1.80	1.93	1.83	1.17	1.28	1.95	1.93	1.47	1.48	1.95	1.50	1.89	1.50	1.47	1.70	1.67	1.42	1.98	1.96
GZ-ZY	1.70	1.51	1.73	1.30	1.30	1.52	1.90	2.03	1.48	1.54	1.84	1.74	1.89	1.74	1.31	1.68	1.74	1.74	1.99	2.00
HN-DX	1.91	1.86	1.90	1.75	1.83	1.47	1.69	1.84	1.82	1.78	1.91	1.56	1.75	1.83	1.70	1.61	1.50	1.70	1.83	1.94
JX-DX	1.33	1.24	1.79	1.04	1.39	1.04	1.56	1.79	1.56	1.01	1.01	1.04	1.39	1.04	1.04	1.04	1.04	1.04	1.24	1.79
JX-WN	1.70	1.81	1.92	1.50	1.42	1.83	1.83	1.96	1.45	1.77	1.92	1.61	1.89	1.70	1.50	1.36	1.50	1.70	1.93	1.93
JX-WY	1.63	1.85	1.81	1.10	1.10	1.10	1.83	2.01	1.13	1.35	1.48	1.10	1.10	1.10	1.10	1.79	1.10	1.10	1.81	1.83
JX-WZ	1.99	1.73	1.59	1.55	1.15	1.28	1.59	1.85	1.28	1.78	1.88	1.64	1.61	1.61	1.89	1.77	1.70	1.70	1.96	1.91
JX-YZ	1.72	1.50	1.93	1.73	1.56	1.07	2.04	2.02	1.60	1.78	1.81	1.75	1.50	1.83	1.70	1.70	1.70	1.70	1.98	1.87
SC-HJ	1.85	0.98	1.82	1.89	1.52	1.30	1.86	1.86	1.70	1.78	1.77	1.43	1.74	1.68	1.52	1.00	1.74	1.22	1.89	1.97
SC-KJ	1.93	1.79	1.89	1.70	1.22	1.36	1.97	1.81	0.87	1.29	1.67	1.75	1.70	1.75	1.70	1.70	1.61	1.75	1.91	1.84
SC-LS	1.82	1.78	1.46	1.58	1.27	1.15	1.81	1.83	1.43	1.51	1.94	1.52	1.58	1.74	1.74	1.21	1.68	1.43	1.73	1.80
YN-LC	1.89	1.74	1.46	1.33	1.05	0.95	1.74	1.74	0.35	0.35	1.68	1.33	0.95	1.33	0.95	1.33	1.61	1.61	1.68	1.74
YN-MG	2.00	1.86	1.93	1.67	1.26	1.32	1.87	1.76	1.29	1.35	1.87	1.61	1.47	1.70	1.70	1.89	1.70	1.19	1.90	1.79
YN-MH	1.83	1.90	1.83	1.70	1.70	1.83	1.99	1.91	1.60	1.75	1.81	1.36	1.70	1.75	1.70	1.19	1.83	1.61	1.77	1.82
YN-MJ	1.64	1.49	1.82	1.64	1.70	1.70	2.02	1.97	1.40	1.64	1.87	1.42	1.50	1.70	1.70	1.56	1.83	1.70	1.93	1.99
YN-SM	1.45	1.42	1.84	1.55	1.28	0.80	1.71	1.62	1.49	1.45	1.99	1.75	1.75	1.48	1.55	1.28	1.48	1.48	1.43	1.81
YN-XC	1.71	1.87	1.96	1.28	1.15	1.28	1.94	1.87	0.44	0.44	1.96	1.75	1.48	1.75	1.28	1.15	1.75	1.35	1.73	1.76
ZJ-JS	1.68	1.50	1.82	—	—	—	1.83	1.88	1.82	1.54	1.85	1.68	1.74	1.52	1.74	1.43	1.52	1.31	1.90	1.92
ZJ-LD	1.86	1.91	1.78	1.75	1.55	1.55	1.93	1.59	1.56	1.93	1.76	1.28	1.83	1.75	1.47	1.89	1.75	1.50	1.76	1.85
ZJ-LY	1.39	1.63	1.81	1.33	1.01	1.33	1.75	1.67	1.68	1.74	1.77	0.96	1.15	1.08	1.55	1.35	1.48	1.15	1.79	1.69
ZJ-QY	1.66	1.83	1.61	1.56	1.33	1.56	1.84	1.90	1.81	1.83	1.77	1.74	1.74	1.74	1.46	1.68	1.74	1.52	1.97	1.91
ZJ-SC	1.69	1.82	1.83	1.33	1.56	1.33	1.77	1.79	1.89	1.73	1.91	1.70	1.50	1.36	1.70	1.70	1.55	1.64	1.71	1.92
mean	1.71	1.69	1.78	1.50	1.36	1.35	1.82	1.85	1.44	1.54	1.81	1.53	1.58	1.54	1.48	1.48	1.58	1.51	1.82	1.84

BD, Basal diameter; CD, Crown diameter; TH, Diameter height; EOC, Essential oil content; EOY, Essential oil yield; EOY/BD, Essential oil yield/ Basal diameter; FPKR, Fresh pulp kernel ratio; FFPR, Fresh fruit pulp rate; FWC, Water content of fruit; FY, fresh fruit yield; FY/BD, Fruit yield/ Basal diameter; TFW, Thousand-grain fresh weight; TDW, Thousand-grain dry weight; LA, Leaf area; LL, Leaf length; LW, Leaf width; LP, Leaf perimeter; PL, Petiole length; LSI, Leaf shape index; LV, Number of leaf veins; Shannon─Wiener index (*H′*) was calculated by the method as described by Hamil et al. (2021).

Table S5. Nested variance analysis for phenotypic traits of *L. cubeba*

Traits	Mean square			*F* value	
	Within provenances	Among provenances	Random errors	Within provenances	Among provenances
BD (mm)	3511.51	11418.89	1078.60	3.26**	10.59**
CD (m^2)	76.43	199.82	38.26	2.00**	5.22**
TH (m)	5.87	42.01	2.44	2.41**	17.25**
EOC (%)	0.41	2.93	0.36	1.14	8.19**
EOY (g)	15542.03	29133.90	11415.32	1.36	2.55**
EOY/BD ($g.cm^{-1}$)	307.45	536.28	158.50	1.94**	3.38**
FPKR	1.17	18.37	1.01	1.16	18.24**
FFPR (%)	25.95	272.60	18.97	1.37	14.37**
FY (kg)	16.99	30.98	7.71	2.20**	4.02**
FY/BD ($g.cm^{-1}$)	0.18	0.46	0.11	1.62*	4.21**
LA (cm^2)	13.26	342.11	26.56	0.50	12.88**
LL (cm)	1.72	19.75	1.74	0.99	11.32**
LP (cm)	0.07	0.35	0.04	1.70*	9.02**
PL (cm)	15.57	367.19	9.48	1.64*	38.72**
LSI	0.37	1.09	0.22	1.66*	4.87**
LV	6.94	33.93	6.33	1.10	5.36**
LW (cm)	0.15	3.31	0.25	0.60	13.31**
TDW (g)	84.51	1524.26	105.52	0.80	14.44**
TFW (g)	505.56	7232.00	599.98	0.84	12.05**
FWC (%)	35.82	1094.44	35.60	1.01	30.74**

BD, Basal diameter; CD, Crown diameter; TH, Diameter height; EOC, Essential oil content; EOY, Essential oil yield; EOY/BD, Essential oil yield/ Basal diameter; FPKR, Fresh pulp kernel ratio; FFPR, Fresh fruit pulp rate; FWC, Water content of fruit; FY, fresh fruit yield; FY/BD, Fruit yield/ Basal diameter; TFW, Thousand-grain fresh weight; TDW, Thousand-grain dry weight; LA, Leaf area; LL, Leaf length; LW, Leaf width; LP, Leaf perimeter; PL, Petiole length; LSI, Leaf shape index; LV, Number of leaf veins; Asterisks indicate significance level (*$P \leq 0.05$,**$P \leq 0.01$).

Table S6. Multiple comparison on leaf and growth characteristics between provenances of *L. cubeba*

SN	BD (mm)	CD (m^2)	TH (m)	LA (cm^2)	LSI	LL (cm)	LP (cm)	LW (cm)	LV	PL (cm)
AH-DZ	46.53±7.64 jkl	3.68±0.52 fg	4.68±0.36 hijk	10.09±0.78 ijkl	3.37±0.17 cdefgh	7.12±0.37 ijklm	18.44±0.71 ijkl	2.16±0.12 ghi	21±0.19 cdefgh	0.91±0.06 ghij
AH-JX	116.53±11.78 b	3.87±0.68 fg	2.31±0.08 o	11.75±0.60 hijk	3.61±0.06 bcde	7.87±0.18 ghijk	21.30±0.45 efghi	2.22±0.06 ghi	22±1.03 bcdef	1.27±0.05 cde
AH-QM	42.98±7.82 jkl	4.23±0.55 fg	5.54±0.27 efgh	13.78±0.72 ghij	3.79±0.15 bc	8.72±0.31 efg	22.25±0.68 def	2.33±0.09 fghi	23.8±0.73 abcd	1.01±0.06 fghi
AH-QY	42.56±6.32 jkl	7.71±1.56 bcdef	7.11±0.53 bc	15.73±0.62 efghi	3.42±0.08 bcdefgh	8.98±0.26 defg	23.19±0.46 cdef	2.65±0.03 cdefg	24.00±1.35 abc	0.93±0.12 fghij
AH-XN	68.35±9.98 fghi	4.62±0.64 fg	4.16±0.26 jklm	13.27±1.12 ghij	3.55±0.06 bcde	8.11±0.38 fghij	21.30±0.84 efghi	2.30±0.10 fghi	23.00±0.49 bcd	1.09±0.07 efg
FJ-CT	79.86±9.05 defg	5.14±0.83 defg	6.02±0.26 cdefg	11.20±1.9 hijk	3.40±0.16 bcdefgh	7.23±0.57 hijklm	20.28±1.20 efghij	2.20±0.22 ghi	19.67±0.56 fgh	1.10±0.09 efg
FJ-JO	50.62±4.67 ijk	4.18±0.56 fg	4.47±0.24 ijkl	14.16±1.51 fghij	3.44±0.07 bcdef	8.45±0.51 efgh	21.77±1.27 defg	2.47±0.14 efg	20.89±1.05 defgh	0.92±0.06 ghij
FJ-LC	48.56±3.66 jk	4.53±0.58 fg	3.91±0.20 jklm	7.63±0.72 kl	2.85±0.08 ijk	5.49±0.35 n	16.17±0.96 l	1.93±0.11 ij	17.00±0.79 i	0.78±0.06 jkl
FJ-SX	54.43±4.69 hij	6.5±0.81 def	4.16±0.22 jklm	13.53±0.70 ghij	3.35±0.09 defgh	8.04±0.20 ghij	21.59±0.75 defgh	2.43±0.08 efg	21.00±0.42 defgh	0.84±0.05 ijk
FJ-ZH	60.57±8.71 ghij	9.76±3.14 abcd	7.34±0.48 b	13.08±1.06 ghij	3.57±0.18 bcde	8.12±0.29 fghij	21.66±0.84 defgh	2.34±0.13 fghi	20.30±0.60 efgh	0.91±0.05 ghij
GX-GY	80.45±7.92 def	11.51±1.76 ab	6.10±0.30 cdefg	18.70±2.94 def	3.38±0.25 bcdefgh	9.36±0.67 cdef	24.44±1.60 cd	2.89±0.30 bcde	23.29±0.64 bcd	1.06±0.07 efgh
GZ-BL	91.64±13.59 cd	12.90±2.55 a	4.78±0.33 hij	5.60±0.31 l	3.66±0.17 bcde	5.48±0.22 n	6.07±0.22 o	1.51±0.04 j	22.14±0.59 bcdef	0.63±0.03 l
GZ-TJ	89.20±7.02 cde	11.57±1.65 ab	6.21±0.25 cdef	14.26±0.96 fghi	3.44±0.12 bcdef	8.48±0.27 efgh	9.47±0.29 no	2.50±0.11 efg	23.60±0.76 bcd	1.05±0.06 fgh
GZ-ZF	68.64±7.93 fghi	7.61±1.81 cdef	4.47±0.64 ijk	7.67±1.13 jkl	3.22±0.17 efghi	5.90±0.43 mn	6.56±0.47 o	1.87±0.16 ij	21.00±0.65 defgh	0.67±0.05 kl
GZ-ZJ	64.12±6.57 fghij	10.51±1.45 abc	5.30±0.40 ghi	8.85±1.96 jkl	3.35±0.19 defgh	6.03±0.53 mn	6.87±0.62 o	1.92±0.25 ij	20.20±0.96 fgh	0.89±0.10 hij
GZ-ZY	57.60±6.70 hij	5.91±1.48 defg	3.5±0.33 mn	32.23±3.76 a	2.66±0.09 jk	11.12±0.59 ab	12.34±0.63 m	4.25±0.30 a	20.00±0.80 fgh	1.25±0.08 de
HN-DX	58.00±4.98 hij	4.42±0.49 fg	5.28±0.24ghi	15.88±1.13 efgh	3.54±0.06 bcde	9.06±0.35 defg	23.34±0.87 cde	2.58±0.09 defg	22.60±0.65 bcd	1.27±0.05 cde
JX-DX	42.03±10.07 jkl	4.16±1.71 fg	3.21±0.50 mno	17.86±1.70 defg	3.53±0.30 bcdef	9.85±0.40 bcde	10.98±0.41 mn	2.84±0.21 bcdef	21.50±1.32 cdefgh	1.17±0.01 def
JX-WN	60.88±5.60 ghij	9.19±1.04 bcd	4.11±0.26 jklm	15.80±1.38 efgh	3.02±0.13 fghij	8.37±0.35 efgh	9.29±0.34 no	2.79±0.14 cdef	23.40±0.95 bcd	1.03±0.05 fgh
JX-WY	51.79±4.39 hijk	5.63±0.62 defg	4.37±0.24 ijklm	14.21±1.68 fghij	4.04±0.61 ab	9.13±0.42 defg	10.34±0.48 mno	2.36±0.28 fghi	24.00±1.15 abcd	1.33±0.11 bcde
JX-WZ	32.53±1.99 kl	5.51±0.68 defg	3.78±0.32 klmn	17.24±1.47 efg	3.36±0.26 cdefgh	9.27±0.35 def	10.24±0.35 mno	2.77±0.18 cdef	24.00±1.33 abcd	1.09±0.06 efg
JX-YZ	27.19±2.32 l	3.33±0.67 fg	2.86±0.15 no	17.53±1.56 defg	2.90±0.11 hijk	8.56±0.42 efgh	9.46±0.46 no	2.98±0.17 bcd	19.40±0.75 gh	1.04±0.07 fgh
SC-HJ	80.73±3.36 def	10.93±2.94 abc	6.58±0.32 bcd	31.65±4.11 a	2.94±0.13 ghijk	11.54±0.74 a	30.64±1.95 a	4.03±0.31 a	23.22±0.92 bcd	1.46±0.06 ab
SC-KJ	51.43±3.78 ijk	10.92±1.30 abc	3.54±0.16 lmn	24.95±1.63 bc	3.35±0.09 defgh	11.07±0.42 ab	27.85±1.00 ab	3.33±0.10 b	23.30±0.78 bcd	1.14±0.07 ef
SC-LS	56.33±4.39 hij	8.48±0.99 bcde	5.31±0.36 fghi	25.59±2.93 b	2.55±0.14 k	9.61±0.48 cde	25.25±1.31 bc	3.89±0.30 a	21.67±0.99 cdefg	1.37±0.12 bcd
YN-LC	66.61±3.84 fghij	10.15±1.09 abcd	6.82±0.54 bcd	22.82±2.73 bcd	3.43±0.24 bcdefg	10.79±1.03 abc	28.79±2.15 a	3.16±0.15 bc	26.40±2.29 a	1.60±0.19 a
YN-MG	63.38±5.27 fghij	9.85±1.09 abcd	6.73±0.42 bcd	9.32±1.92 jkl	3.37±0.10 cdefgh	6.57±0.58 lmn	18.19±1.31 jkl	1.97±0.18 hij	18.80±0.66 hi	0.88±0.07 hij
YN-MH	71.60±5.20 efgh	9.50±0.82 abcd	5.9±0.50 defg	10.82±0.66 ijk	3.49±0.06 bcdef	7.41±0.25 hijkl	19.99±0.62 fghij	2.14±0.07 ghi	22.50±0.81 bcde	1.01±0.05 fghi
YN-MJ	105.44±9.10 bc	6.73±1.13 def	7.06±0.34 bc	13.79±1.69 ghij	3.36±0.22 cdefgh	7.72±0.59 ghijkl	21.18±1.46 efghi	2.37±0.17 fgh	22.20±0.47 bcdef	1.04±0.05 fgh
YN-SM	147.51±22.08 a	11.75±1.77 ab	8.70±0.83 a	15.31±2.33 efghi	3.18±0.12 efghi	8.15±0.76 fghij	22.98±2.08 cdef	2.56±0.19 defg	24.43±0.53 ab	1.36±0.09 bcd
YN-XC	70.30±5.45 efghi	9.32±1.13 abcd	5.98±0.37 cdefg	20.14±1.61 cde	3.66±0.16 bcde	10.34±0.36 abcd	28.68±1.06 a	2.87±0.17 bcde	26.14±0.99 a	1.45±0.10 abc
ZJ-JS	30.89±3.69 kl	4.85±1.25 efg	4.6±0.38 hijk	14.12±1.50 fghij	3.44±0.23 bcdef	8.32±0.33 efghi	16.35±2.16 kl	2.51±0.19 efg	21.89±1.01 cdef	1.14±0.04 ef
ZJ-LD	61.89±6.45 fghij	12.80±1.74 a	6.57±0.41 bcd	9.51±0.60 jkl	3.28±0.11 efgh	6.84±0.32 klm	18.89±0.82 hijkl	2.11±0.06 ghi	19.20±0.61 hi	0.89±0.05 hij
ZJ-LY	56.88±9.44 hij	7.20±1.69 cdef	6.49±0.38 bcde	9.64±1.38 ijkl	3.76±0.15 bcd	7.34±0.54 hijkl	20.13±1.46 fghij	1.96±0.14 hij	24.00±1.00 abcd	1.07±0.07 efgh
ZJ-QY	46.27±2.97 jkl	5.35±0.41 defg	5.85±0.30 defg	10.13±0.85 ijkl	4.44±0.42 a	8.19±0.54 fghi	21.73±1.47 defgh	1.91±0.12 ij	23.67±1.13 abcd	1.12±0.05 ef
ZJ-SC	53.12±7.38 hij	2.68±0.41 g	3.32±0.23 mn	8.52±0.68 jkl	3.77±0.14 bcd	6.98±0.33 jklm	18.96±0.83 ghijk	1.88±0.08 ij	21.20±0.76 cdefgh	1.07±0.06 efg

BD, Basal diameter; CD, Crown diameter; TH, Diameter height; LA, Leaf area; LL, Leaf length; LW, Leaf width; LP, Leaf perimeter; PL, Petiole length; LSI, Leaf shape index; LV, Number of leaf veins;

Table S7. Multiple comparison on fruit characteristics between provenances of *L. cubeba*

SN	TFW (g)	TDW (g)	FWC (%)	FFPR (%)	FPKR	FY (kg)	FY/BD ($g.cm^{-1}$)	EOC (%)	EOY (g)	EOY/BD ($g. cm^{-1}$)
AH-DZ	126.11±6.16 ghijk	55.16±2.54 cdefg	55.53±2.10 mno	76.33±0.87 hij	3.32±0.16 hijk	0.58±0.11 efg	0.17±0.04 def	2.85±0.25 defgh	27.48±4.84 d	6.81±2.06 cdef
AH-JX	140.19±8.21 efg	66.47±3.75 a	51.59±2.00 p	71.95±1.57 lm	2.83±0.21 klm	0.38±0.06 efg	0.04±0.01 f	3.49±0.25 bc	22.03±2.14 d	1.68±0.31 f
AH-QM	123.58±5.36 ijk	50.74±1.98 efghij	58.45±1.23 klmn	79.01±0.85 efgh	3.95±0.23 efgh	0.43±0.06 efg	0.17±0.03 def	3.51±0.14 bc	19.33±2.70 d	5.10±1.13 ef
AH-QY	127.17±5.29 ghijk	51.91±2.12 defghij	58.84±1.62 jklmn	79.34±1.34 defgh	4.07±0.40 efgh	0.21±0.06 fg	0.05±0.01 f	3.58±0.27 abc	14.71±4.33 d	2.84±1.13 f
AH-XN	115.94±4.03 klm	47.16±1.72 ijkl	58.9±1.32 jklm	79.28±0.96 efgh	4.16±0.32 defg	0.41±0.10 efg	0.05±0.01 f	3.83±0.45 ab	53.92±9.02 cd	4.62±1.14 ef
FJ-CT	105.88±3.32 lmn	39.67±1.52 no	62.37±1.27 fghij	78.97±0.99 efgh	3.91±0.21 efgh	0.83±0.14 defg	0.10±0.01 ef	3.75±0.50 abc	42.61±14.87 cd	4.23±1.28 ef
FJ-JO	122.05±4.68 jkl	41.57±1.33 lmno	65.52±0.97 def	80.02±1.16 defg	4.27±0.25 cde	0.48±0.08 efg	0.09±0.01 ef	3.79±0.15 ab	35.71±5.43 d	4.49±0.44 ef
FJ-LC	99.18±3.00 n	44.54±1.80 jklmn	55.07±1.20 no	75.66±0.87 ij	3.24±0.16 ijk	0.40±0.08 efg	0.08±0.01 f	3.89±0.10 ab	51.89±7.29 cd	7.97±1.53 cdef
FJ-SX	125.48±3.22 hijk	49.52±1.78 ghij	60.32±1.34 hijklm	79.02±0.76 efgh	3.92±0.18 efgh	0.50±0.09 efg	0.09±0.02 ef	2.84±0.21 efgh	20.27±4.68 d	3.47±0.82 f
FJ-ZH	128.04±5.16 ghijk	49.31±2.27 ghijk	61.2±1.55 ghijkl	77.99±1.24 fghi	3.82±0.32 efghi	0.72±0.33 efg	0.10±0.03 ef	—	—	—
GX-GY	136.89±4.85 efghi	56.75±2.03 cde	58.17±1.29 lmn	77.73±0.88 ghi	3.63±0.17 ghij	1.85±0.44 cdef	0.23±0.04 def	3.29±0.18 bcde	114.63±30.82 bcd	13.16±3.28 cdef
GZ-BL	178.86±7.14 a	64.58±1.86 ab	63.40±0.83 efgh	79.56±0.54 defg	3.97±0.14 efgh	0.9±0.30 defg	0.08±0.02 ef	2.79±0.14 efgh	55.51±21.08 cd	3.82±1.20 ef
GZ-TJ	116.45±4.57 klm	50.45±2.01 fghij	56.42±0.90 1mn	75.28±0.64 ijk	3.11±0.11 jkl	2.59±0.55 bcde	0.27±0.05 cde	3.27±0.23 bcde	105.43±22.22 bcd	9.66±1.93 cdef
GZ-ZF	146.76±4.92 def	52.61±2.34 defghi	63.87±1.54 efg	80.31±0.76 def	4.23±0.19 def	3.05±0.95 abc	0.36±0.07 cd	2.73±0.11 efghi	118.48±65.41 bcd	12.47±3.82 cdef
GZ-ZJ	145.4±5.54 def	49.76±1.46 fghij	65.32±0.80 ef	80.04±0.64 defg	4.14±0.17 efg	1.40±0.37 defg	0.21±0.04 def	2.63±0.25 fghi	52.57±26.43 cd	6.93±2.26 cdef
GZ-ZY	153.18±4.29 cde	57.80±1.58 cd	62.07±0.72 fghij	77.74±0.84 ghi	3.64±0.17 ghij	4.31±1.20 a	0.57±0.14 ab	2.44±0.17 ghij	135.51±42.94 bcd	17.28±5.42 cd
HN-DX	120.36±5.02 jkl	56.70±2.96 cde	52.90±1.50 op	74.62±0.94 jk	3.10±0.19 jkl	0.69±0.07 efg	0.13±0.01 ef	4.22±0.21 a	38.24±4.18 d	6.17±1.00 def
JX-DX	108.62±7.50 klmn	43.06±3.39 klmno	60.49±0.62 ghijklm	78.82±1.13 efghi	3.79±0.26 efghij	2.88±0.84 abcde	0.74±0.31 a	3.41±0.11 bcde	137.70±19.76 bcd	34.67±15.00 ab
JX-WN	121.74±6.03 jkl	48.1±1.85 hijk	59.56±1.32 ijklm	76.22±0.90 hij	3.36±0.18 hijk	2.88±0.61 abcde	0.43±0.06 bc	3.29±0.21 bcde	122.64±37.95 bcd	16.58±2.91 cde
JX-WY	122.3±4.35 ijkl	42.8±1.94 klmno	64.87±1.24 efg	81.89±1.09 cde	4.80±0.37 cd	0.52±0.17 efg	0.11±0.03 ef	2.55±0.05 fghij	30.76±17.56 d	5.56±2.43 def
JX-WZ	121.57±5.44 jkl	37.61±2.08 o	69.05±1.00 bcd	82.10±0.86 cd	4.89±0.38 c	0.64±0.17 efg	0.19±0.04 def	3.00±0.31 cdefg	21.76±5.13 d	6.72±1.32 cdef
JX-YZ	123.56±3.53 ijk	45.97±1.46 jklm	62.69±0.86 fghi	78.33±0.51 fghi	3.68±0.11 fghij	0.47±0.09 efg	0.16±0.02 ef	3.42±0.27 bcd	24.70±4.37 d	8.80±1.21 cdef
SC-HJ	140.93±3.77 efg	53.5±1.24 defgh	61.82±0.70 ghijk	78.23±0.77 fghi	3.73±0.18 efghij	1.08±0.14 defg	0.13±0.02 ef	3.26±0.13 bcde	55.09±10.03 cd	6.14±1.06 def
SC-KJ	125.50±6.79 hijk	70.26±2.64 a	42.03±2.25 r	70.46±1.29 m	2.52±0.14 m	2.86±1.01 abcde	0.45±0.13 abc	4.17±0.24 a	269.09±103.14 a	39.76±12.92 a
SC-LS	139.17±5.69 efgh	52.81±2.14 defghi	61.44±1.39 ghijkl	77.58±0.92 ghi	3.64±0.20 fghij	0.87±0.20 defg	0.14±0.02 ef	2.78±0.20 efgh	39.86±13.49 d	5.60±1.72 def
YN-LC	155.33±7.38 bcde	38.13±4.17 o	75.84±1.89 a	87.49±0.77 a	7.22±0.45 a	3.04±2.33 abcd	0.49±0.37 abc	2.44±0.34 ghij	127.8±105.33 bcd	20.72±16.61 bc
YN-MG	116.27±3.93 klm	38.34±1.48 o	66.74±1.16 cde	84.09±0.82 bc	5.58±0.28 b	1.89±0.62 cde	0.27±0.08 cde	1.90±0.27 jk	76.66±35.69 cd	9.85±4.00 cdef
YN-MH	140.33±5.39 efg	41.17±2.45 mno	70.90±0.97 b	84.68±0.65 abc	5.78±0.26 b	1.48±0.29 cdefg	0.20±0.03 def	1.75±0.10 k	48.35±10.60 cd	5.80±1.23 def
YN-MJ	171.28±4.81 ab	43.51±1.35 klmno	74.58±0.37 a	76.37±0.90 hij	3.39±0.17 hijk	2.61±0.38 bcde	0.25±0.03 cdef	2.86±0.11 defg	125.74±17.01 bcd	9.92±0.88 cdef
YN-SM	160.26±4.43 bcd	43.29±2.77 klmno	73.05±1.34 ab	86.03±1.23 ab	6.79±0.55 a	4.43±1.58 a	0.25±0.09 cdef	2.24±0.11 hijk	193.91±62.51 ab	10.49±4.04 cdef
YN-XC	165.66±6.92 abc	49.89±2.36 fghij	69.79±0.87 bc	77.93±1.08 fghi	3.7±0.22 efghij	3.73±2.59 ab	0.48±0.31 abc	2.11±0.14 ijk	153.57±103.61 bc	19.70±12.47 bc
ZJ-JS	104.77±3.68 mn	45.41±1.97 jklmn	56.35±1.73 mno	77.65±0.95 ghi	3.64±0.21 ghij	0.11±0.01 g	0.05±0.01 f	—	—	—
ZJ-LD	112.79±7.68 klmn	54.25±3.41 defg	51.52±0.67 p	72.99±1.39 kl	2.86±0.15 klm	0.54±0.08 efg	0.09±0.01 ef	3.51±0.28 bc	35.84±3.94 d	4.58±0.81 ef
ZJ-LY	134.51±5.48 fghij	70.84±2.52 a	47.00±1.06 q	71.71±0.62 lm	2.56±0.08 lm	0.64±0.13 efg	0.12±0.02 ef	3.65±0.28 abc	9.48±2.21 d	2.38±0.57 f
ZJ-QY	124.50±3.97 ijk	55.57±1.86 cdef	55.28±0.75 mno	75.21±0.64 ijk	3.10±0.12 jklm	0.40±0.05 efg	0.09±0.01 ef	3.13±0.21 bcdef	21.24±1.40 d	4.19±0.54 ef
ZJ-SC	133.94±4.06 fghij	60.35±2.54 bc	54.96±1.46 no	74.70±0.97 jk	3.11±0.18 jkl	0.27±0.04 fg	0.06±0.01 f	3.38±0.18 bcde	18.05±2.84 d	3.60±1.23 ef

EOC, Essential oil content; EOY, Essential oil yield; EOY/BD, Essential oil yield/ Basal diameter; FPKR, Fresh pulp kernel ratio; FFPR, Fresh fruit pulp rate; FWC, Water content of fruit; FY, fresh fruit yield; FY/BD, Fruit yield/ Basal diameter; TFW, Thousand-grain fresh weight; TDW, Thousand-grain dry weight;

Table S8. The result of path analysis for LA of *L. cubeba*

Factors	Path coefficient (LA - response factors)											
	Direct path coefficient	Indirect path coefficient										
		STPC	SBD	SMaxWHC	STOC	STNC	Longi	AARH	SMinWHC	AAMinT	AAR	STKC
STPC	-0.374		-0.055	-0.023	-0.012	-0.001	0.084	-0.007	-0.024	-0.022	0.008	-0.023
SBD	0.214	0.096		0.031	0.010	0.001	0.101	0.022	0.032	0.038	0.009	-0.007
SMaxWHC	-0.046	-0.187	-0.144		-0.013	-0.001	0.055	0.011	-0.059	-0.039	0.009	-0.009
STOC	-0.020	-0.226	-0.106	-0.029		-0.002	0.079	-0.006	-0.031	-0.023	0.008	-0.018
STNC	-0.002	-0.185	-0.110	-0.023	-0.017		0.029	-0.020	-0.022	-0.026	0.006	0.010
Longi	-0.268	0.118	-0.080	0.009	0.006	0.000		-0.025	0.021	0.010	-0.038	0.018
AARH	0.086	0.029	0.055	-0.006	0.001	0.000	0.078		-0.014	-0.023	0.011	-0.027
SMinWHC	-0.066	-0.137	-0.105	-0.041	-0.009	-0.001	0.085	0.019		-0.036	0.013	-0.017
AAMinT	0.108	0.078	0.075	0.017	0.004	0.000	-0.025	-0.019	0.022		-0.010	-0.011
AAR	-0.054	0.058	-0.037	0.008	0.003	0.000	-0.191	-0.017	0.016	0.020		0.001
STKC	0.149	0.058	-0.010	0.003	0.002	0.000	-0.032	-0.015	0.008	-0.008	0.000	

Longi, Longitude; AAMinT, annual average minimum temperature; AARH, annual average relative humidity; AAR, annual average rainfall; SBD, Soil bulk density; SMaxWHC, Soil Maximum water holding capacity; SMinWHC, Soil Minimum water holding capacity; STOC, Soil total carbon content; STNC, Soil total nitrogen content; STPC, Soil total phosphorus content; STKC, Soil total potassium content.

Table S9. The result of path analysis for LSI of *L. cubeba*

Factors	Path coefficient (LSI - response factors)										
	Direct path coefficient	Indirect path coefficient									
		SBD	STPC	SpH	STNC	STOC	AARH	AAR	Longi	SMaxWHC	SP
SBD	-0.261		-0.049	0.009	-0.031	-0.001	-0.012	-0.037	-0.026	-0.011	-0.004
STPC	0.190	0.067		0.061	0.030	0.001	0.004	-0.033	-0.022	0.008	-0.009
SpH	0.259	-0.009	0.045		0.008	0.000	0.011	-0.015	-0.001	-0.003	0.007
STNC	0.061	0.134	0.094	0.033		0.002	0.011	-0.025	-0.007	0.008	-0.003
STOC	0.002	0.129	0.115	0.020	0.052		0.003	-0.032	-0.020	0.010	-0.009
AARH	-0.047	-0.067	-0.015	-0.062	-0.015	0.000		-0.043	-0.020	0.002	-0.015
AAR	0.214	0.045	-0.030	-0.018	-0.007	0.000	0.009		0.049	-0.003	0.011
Longi	0.069	0.098	-0.060	-0.005	-0.007	-0.001	0.014	0.152		-0.003	0.021
SMaxWHC	0.016	0.175	0.095	-0.042	0.030	0.001	-0.006	-0.036	-0.014		-0.019
SP	-0.031	-0.031	0.057	-0.060	0.006	0.001	-0.023	-0.077	-0.046	0.010	

Longi, Longitude; AARH, annual average relative humidity; AAR, annual average rainfall; SBD, Soil bulk density; SpH, Soil pH; SMaxWHC, Soil Maximum water holding capacity; STOC, Soil total carbon content; STNC, Soil total nitrogen content; STPC, Soil total phosphorus content;

Table S10. The result of path analysis for TFW of *L. cubeba*

Factors	Path coefficient (TFW - response factors)																	
	Direct path coefficient	Indirect path coefficient																
		AAR	Longi	Ele	AAMinT	BD	AAT	SWC	PL	AAMaxT	Lati	LA	LV	SMinWHC	SP	CD	LP	LL
AAR	0.069		0.029	-0.292	-0.058	-0.016	0.010	0.018	-0.001	0.010	0.006	-0.021	-0.001	0.035	0.001	-0.007	-0.034	-0.004
Longi	0.041	0.049		-0.368	-0.030	-0.021	0.004	0.026	-0.012	0.002	0.016	-0.023	0.001	0.046	0.001	-0.008	-0.017	-0.013
Ele	0.477	-0.042	-0.032		0.091	0.024	-0.008	-0.036	-0.001	-0.004	-0.016	-0.011	0.000	-0.091	-0.001	0.006	-0.016	-0.030
AAMinT	-0.322	0.012	0.004	-0.135		-0.022	0.026	0.019	0.011	0.017	-0.004	0.022	0.000	0.049	0.000	-0.002	0.093	0.037
BD	0.093	-0.012	-0.009	0.124	0.075		-0.005	-0.001	0.005	-0.002	-0.005	-0.012	0.000	-0.005	0.000	0.012	0.017	-0.022
AAT	0.028	0.025	0.006	-0.128	-0.299	-0.017		0.021	0.012	0.022	-0.006	0.012	-0.001	0.047	0.000	-0.004	0.092	0.031
SWC	-0.053	-0.024	-0.020	0.326	0.114	0.002	-0.011		-0.006	-0.007	-0.011	-0.014	0.000	-0.114	-0.001	0.006	-0.068	-0.035
PL	0.068	-0.001	-0.007	-0.004	-0.051	0.007	0.005	0.005		0.004	0.000	0.067	-0.004	0.012	0.000	-0.001	0.107	0.099
AAMaxT	0.023	0.030	0.004	-0.074	-0.243	-0.008	0.026	0.017	0.012		-0.009	0.000	-0.001	0.030	0.000	-0.004	0.080	0.020
Lati	0.023	0.017	0.028	-0.329	0.060	-0.021	-0.007	0.025	0.000	-0.009		0.013	0.000	0.059	0.001	-0.006	0.002	0.024
LA	0.110	-0.013	-0.009	-0.048	-0.066	-0.010	0.003	0.007	0.042	0.000	0.003		-0.003	0.030	0.000	0.000	0.099	0.136
LV	-0.011	0.004	-0.004	0.013	-0.002	-0.003	0.002	0.002	0.025	0.002	0.000	0.035		0.012	0.000	-0.001	0.081	0.069
SMinWHC	-0.146	-0.017	-0.013	0.296	0.107	0.003	-0.009	-0.041	-0.005	-0.005	-0.009	-0.023	0.001		-0.001	0.004	-0.052	-0.041
SP	-0.002	-0.025	-0.027	0.275	0.023	0.007	-0.003	-0.038	0.003	-0.001	-0.015	0.005	0.000	-0.098		0.006	-0.036	-0.011
CD	0.025	-0.020	-0.013	0.120	0.028	0.044	-0.004	-0.012	-0.003	-0.003	-0.005	0.001	0.000	-0.022	0.000		0.001	-0.006
LP	0.202	-0.012	-0.003	-0.037	-0.147	0.008	0.013	0.018	0.036	0.009	0.000	0.054	-0.004	0.037	0.000	0.000		0.091
LL	0.150	-0.002	-0.003	-0.097	-0.079	-0.013	0.006	0.012	0.045	0.003	0.004	0.100	-0.005	0.040	0.000	-0.001	0.122	

Longi, Longitude; Lati, Latitude; Ele, Elevation; AAT, Annual average temperature; AAMaxT, Annual average maximum temperature; AAMinT, annual average minimum temperature; AAR, annual average rainfall; SWC, Soil Water content; SP, Soil porosity; SMinWHC, Soil Minimum water holding capacity; BD, Basal diameter; CD, Crown diameter; LA, Leaf area; LL, Leaf length; LP, Leaf perimeter; PL, Petiole length; LV, Number of leaf veins.

Table S11. The result of path analysis for FFPR of *L. cubeba*

Factors	Path coefficient (FFPR - response factors)														
	Direct path coefficient	Indirect path coefficient													
		Lati	Longi	Ele	SP	SWC	TH	AAR	STOC	AARH	SMinWHC	SMaxWHC	STNC	STPC	SpH	CD
Lati	-0.162		0.179	0.140	0.060	-0.036	0.018	0.011	0.037	-0.004	-0.004	-0.040	0.040	0.007	-0.021	0.006
Longi	-0.478	0.061		-0.241	-0.169	0.058	-0.033	-0.020	-0.044	0.007	0.000	0.083	-0.089	-0.010	0.040	-0.002
Ele	-0.357	0.063	-0.323		-0.189	0.059	-0.034	-0.019	-0.125	0.005	0.006	0.065	-0.052	-0.004	0.038	0.001
SP	0.245	-0.039	0.329	0.276		-0.051	0.047	0.017	0.108	-0.009	-0.001	-0.126	0.138	0.015	-0.058	-0.001
SWC	-0.089	-0.066	0.310	0.238	0.141		0.049	0.011	0.063	-0.005	-0.009	-0.137	0.156	0.004	-0.037	0.009
TH	0.069	-0.042	0.226	0.174	0.168	-0.063		0.012	0.061	-0.009	-0.004	-0.159	0.210	0.013	-0.051	0.012
AAR	0.048	-0.039	0.203	0.142	0.085	-0.020	0.018		0.062	-0.005	0.000	-0.030	0.051	0.010	-0.031	-0.002
STOC	-0.176	0.034	-0.120	-0.254	-0.150	0.032	-0.024	-0.017		0.003	0.004	0.049	-0.043	-0.004	0.019	0.003
AARH	-0.017	-0.034	0.209	0.105	0.124	-0.027	0.036	0.014	0.026		0.001	-0.096	0.162	0.034	-0.074	-0.003
SMinWHC	-0.019	-0.032	-0.011	0.104	0.012	-0.043	0.014	-0.001	0.035	0.001		-0.044	0.032	-0.009	0.010	0.009
SMaxWHC	-0.204	-0.031	0.194	0.113	0.152	-0.060	0.054	0.007	0.043	-0.008	-0.004		0.229	0.013	-0.045	0.006
STNC	0.254	-0.026	0.167	0.073	0.133	-0.055	0.057	0.010	0.030	-0.011	-0.002	-0.184		0.019	-0.061	0.006
STPC	0.039	-0.029	0.122	0.039	0.091	-0.009	0.022	0.013	0.020	-0.015	0.005	-0.067	0.126		-0.060	-0.005
SpH	-0.122	-0.029	0.156	0.112	0.116	-0.027	0.029	0.012	0.027	-0.010	0.001	-0.075	0.127	0.019		-0.009
CD	-0.038	0.024	-0.028	0.007	0.010	0.021	-0.022	0.002	0.012	-0.001	0.005	0.033	-0.041	0.005	-0.029	

Longi, Longitude; Lati, Latitude; Ele, Elevation; AARH, annual average relative humidity; AAR, annual average rainfall; SWC, Soil Water content; SP, Soil porosity; SMinWHC, Soil Minimum water holding capacity; SMaxWHC, Soil Maximum water holding capacity; STNC, Soil total nitrogen content; STPC, Soil total phosphorus content; SpH, Soil PH; STOC, Soil total carbon content; CD, Crown diameter; TH, Diameter height; LL, Leaf length.

Table S12. The result of path analysis for FY of *L. cubeba*

Factors	Path coefficient (FY - response factors)												
	Direct path coefficient	Indirect path coefficient											
		Longi	Lati	Ele	CD	BD	TH	SP	SBD	AAR	FWC	STKC	LL
Longi	-0.403		0.003	0.042	-0.053	-0.051	0.001	0.021	-0.030	0.146	0.032	0.032	-0.008
Lati	0.005	-0.272		0.038	-0.038	-0.052	0.001	0.021	-0.008	0.051	0.034	0.057	0.014
Ele	-0.055	0.311	-0.003		0.042	0.060	-0.001	-0.018	-0.006	-0.126	-0.027	-0.040	-0.018
CD	0.166	0.128	-0.001	-0.014		0.107	-0.002	-0.008	0.002	-0.060	-0.009	-0.006	-0.003
BD	0.229	0.090	-0.001	-0.014	0.078		-0.001	-0.002	-0.001	-0.036	-0.008	-0.075	-0.013
TH	-0.003	0.160	-0.002	-0.019	0.084	0.094		-0.007	-0.006	-0.073	-0.019	-0.040	-0.001
SP	-0.032	0.268	-0.003	-0.032	0.040	0.017	-0.001		0.009	-0.073	-0.028	-0.032	-0.006
SBD	0.079	0.152	0.000	0.004	0.004	-0.004	0.000	-0.004		-0.035	-0.014	-0.012	0.029
AAR	0.205	-0.287	0.001	0.034	-0.049	-0.040	0.001	0.011	-0.014		0.014	0.002	-0.002
FWC	-0.058	0.224	-0.003	-0.025	0.026	0.033	-0.001	-0.016	0.019	-0.048		-0.047	0.003
STKC	0.261	-0.049	0.001	0.009	-0.004	-0.066	0.000	0.004	-0.004	0.002	0.011		0.019
LL	0.089	0.034	0.001	0.011	-0.006	-0.033	0.000	0.002	0.026	-0.006	-0.002	0.055	

Longi, Longitude; Lati, Latitude; Ele, Elevation; AAR, annual average rainfall; STKC, Soil total potassium content; SP, Soil porosity; SBD, Soil bulk density; BD, Basal diameter; CD, Crown diameter; TH, Diameter height; LL, Leaf length; WC, Water content of fruit.

Table S13. The result of path analysis for FY/BD of *L. cubeba*

Factors	Path coefficient (FY/BD - response factors)										
	Direct path coefficient	Indirect path coefficient									
		FY	STKC	Longi	Lati	LL	SBD	CD	SP	LA	LV
FY	0.935		0.018	-0.008	-0.008	0.002	0.003	-0.052	0.002	0.001	0.000
STKC	0.128	0.135		0.003	0.007	0.004	-0.001	0.004	-0.001	0.002	0.000
Longi	0.024	-0.309	0.015		0.020	-0.001	-0.006	0.049	-0.007	-0.003	0.000
Lati	0.030	-0.236	0.028	0.016		0.003	-0.002	0.036	-0.007	0.002	0.000
LL	0.017	0.118	0.027	-0.002	0.005		0.005	0.006	-0.001	0.013	0.001
SBD	0.016	0.160	-0.006	-0.009	-0.003	0.006		-0.004	0.001	0.006	0.000
CD	-0.156	0.313	-0.003	-0.008	-0.007	-0.001	0.000		0.003	0.000	0.000
SP	0.011	0.192	-0.015	-0.016	-0.019	-0.001	0.002	-0.038		0.001	0.000
LA	0.014	0.100	0.021	-0.005	0.004	0.015	0.006	-0.001	0.000		0.001
LV	0.002	0.104	0.001	-0.002	0.000	0.008	0.000	0.005	0.000	0.004	

Longi, Longitude; Lati, Latitude; SP, Soil porosity; SBD, Soil bulk density; STKC, Soil total potassium content; CD, Crown diameter; LA, Leaf area; LL, Leaf length; LV, Number of leaf veins; FY, fresh fruit yield.

Table S14. The result of path analysis for EOC of *L. cubeba*

Factors	Path coefficient (EOC - response factors)																						
	Direct path coefficient	Indirect path coefficient																					
		AAMaxT	AARH	AAR	STOC	SpH	STNC	STPC	SWC	SMaxWHC	SMinWHC	SP	Lati	Longi	Ele	TH	TFW	TDW	FWC	FFPR	FPKR	FY	LL
AAMaxT	0.025		0.021	0.024	0.003	0.008	-0.012	-0.002	0.014	-0.006	0.039	0.008	-0.073	0.010	-0.002	0.001	0.007	0.012	-0.032	0.002	-0.015	0.000	0.008
AARH	-0.055	-0.010		-0.011	0.015	-0.021	-0.036	-0.012	-0.009	0.004	-0.040	-0.065	0.004	-0.031	0.000	0.000	-0.002	0.002	-0.041	0.032	-0.034	-0.001	0.006
AAR	0.055	0.011	0.011		0.033	-0.006	-0.017	-0.025	0.015	-0.005	0.045	0.048	0.047	0.075	-0.006	-0.003	0.012	0.001	0.061	-0.033	0.038	0.004	-0.002
STOC	-0.221	0.000	0.004	-0.008		0.007	0.131	0.095	-0.023	0.019	-0.088	-0.040	-0.083	-0.031	0.005	0.003	-0.004	0.004	-0.059	0.033	-0.038	-0.002	-0.015
SpH	0.086	0.002	0.013	-0.004	-0.017		0.019	0.037	0.013	-0.005	0.030	0.031	0.011	-0.002	0.000	0.000	-0.001	-0.007	0.048	-0.024	0.012	-0.002	0.003
STNC	0.152	-0.002	0.013	-0.006	-0.190	0.011		0.078	-0.014	0.015	-0.061	-0.013	-0.048	-0.011	0.004	0.002	-0.004	0.002	-0.039	0.029	-0.029	-0.001	-0.012
STPC	0.158	0.000	0.004	-0.009	-0.133	0.020	0.075		-0.018	0.015	-0.069	-0.040	-0.062	-0.033	0.005	0.002	-0.003	0.004	-0.047	0.028	-0.040	0.000	-0.018
SWC	-0.043	-0.008	-0.011	-0.019	-0.116	-0.027	0.049	0.066		0.025	-0.146	-0.095	-0.089	-0.051	0.007	0.002	-0.008	0.005	-0.085	0.042	-0.037	-0.003	-0.014
SMaxWHC	0.030	-0.005	-0.007	-0.009	-0.141	-0.014	0.076	0.079	-0.036		-0.168	-0.083	-0.066	-0.021	0.005	0.002	-0.004	0.001	-0.037	0.026	-0.025	0.000	-0.019
SMinWHC	-0.187	-0.005	-0.012	-0.013	-0.104	-0.014	0.050	0.058	-0.033	0.027		-0.090	-0.077	-0.033	0.006	0.001	-0.006	0.002	-0.058	0.031	-0.029	-0.001	-0.016
SP	-0.134	-0.001	-0.027	-0.020	-0.067	-0.020	0.015	0.047	-0.031	0.018	-0.126		-0.123	-0.070	0.006	0.002	-0.006	0.013	-0.127	0.066	-0.071	-0.005	-0.004
Lati	0.189	-0.010	-0.001	0.014	0.097	0.005	-0.039	-0.052	0.020	-0.011	0.076	0.087		0.071	-0.007	-0.004	0.006	-0.017	0.152	-0.060	0.076	0.007	0.010
Longi	0.105	0.002	0.016	0.039	0.065	-0.002	-0.016	-0.050	0.021	-0.006	0.059	0.089	0.128		-0.008	-0.004	0.014	-0.009	0.145	-0.063	0.079	0.009	-0.005
Ele	0.010	-0.004	-0.003	-0.034	-0.112	0.003	0.057	0.075	-0.029	0.016	-0.116	-0.077	-0.130	-0.081		0.003	-0.016	0.003	-0.120	0.039	-0.047	-0.006	-0.012
TH	0.009	0.002	0.001	-0.020	-0.066	0.004	0.040	0.040	-0.011	0.006	-0.027	-0.031	-0.080	-0.042	0.003		-0.003	0.010	-0.084	0.038	-0.053	-0.006	-0.001
TFW	-0.037	-0.004	-0.004	-0.017	-0.025	0.001	0.017	0.014	-0.009	0.003	-0.031	-0.020	-0.033	-0.039	0.004	0.001		-0.023	-0.098	0.042	-0.036	-0.003	0.008
TDW	-0.043	-0.007	0.003	-0.002	0.022	0.014	-0.006	-0.014	0.005	-0.001	0.008	0.041	0.074	0.023	-0.001	-0.002	-0.020		0.144	-0.064	0.076	0.002	0.004
FWC	-0.261	0.003	-0.009	-0.013	-0.050	-0.016	0.023	0.028	-0.014	0.004	-0.042	-0.065	-0.110	-0.058	0.005	0.003	-0.014	0.024		0.120	-0.123	-0.004	0.002
FFPR	0.161	0.000	-0.011	-0.011	-0.046	-0.013	0.028	0.028	-0.011	0.005	-0.036	-0.055	-0.071	-0.041	0.002	0.002	-0.010	0.017	-0.194		-0.159	-0.002	-0.001
FPKR	-0.175	0.002	-0.011	-0.012	-0.048	-0.006	0.025	0.036	-0.009	0.004	-0.031	-0.055	-0.082	-0.047	0.003	0.003	-0.008	0.019	-0.183	0.146		-0.002	0.000
FY	-0.026	0.000	-0.002	-0.009	-0.013	0.008	0.003	0.003	-0.004	0.000	-0.010	-0.027	-0.048	-0.035	0.002	0.002	-0.004	0.003	-0.039	0.014	-0.016		0.008
LL	0.060	0.003	-0.005	-0.002	0.053	0.004	-0.030	-0.047	0.010	-0.010	0.051	0.010	0.031	-0.009	-0.002	0.000	-0.005	-0.003	-0.010	-0.002	0.001	-0.003	

Longi, Longitude; Lati, Latitude; Ele, Elevation; AAMaxT, Annual average maximum temperature; AAR, annual average rainfall; AARH, annual average relative humidity; SWC, Soil Water content; SP, Soil porosity; SpH, Soil pH; SMaxWHC, Soil Maximum water holding capacity; SMinWHC, Soil Minimum water holding capacity; STOC, Soil total carbon content; STNC, Soil total nitrogen content; STPC, Soil total phosphorus content; TH, Diameter height; LL, Leaf length; TFW, Thousand-grain fresh weight; TDW, Thousand-grain dry weight; FPKR, Fresh pulp kernel ratio; FFPR, Fresh fruit pulp rate; FWC, Water content of fruit; FY, fresh fruit yield.

Table S15. The result of path analysis for EOY of *L. cubeba*

Factors	Path coefficient (EOY - response factors)														
	Direct path coefficient	Indirect path coefficient													
		FY	CD	Longi	BD	SBD	SpH	TH	STKC	LL	AAR	LA	Ele	Lati	SP
FY	0.886		0.030	-0.001	-0.011	0.017	0.010	0.000	0.004	-0.001	-0.003	0.000	-0.001	-0.031	0.000
CD	0.091	0.297		-0.001	-0.017	0.003	0.001	0.001	-0.001	0.000	-0.005	0.000	-0.001	-0.028	0.000
Longi	0.004	-0.292	-0.029		0.008	-0.036	-0.002	-0.001	0.004	0.000	0.012	0.001	0.002	0.082	-0.001
BD	-0.037	0.253	0.043	-0.001		-0.002	0.010	0.001	-0.009	0.001	-0.003	0.000	-0.001	-0.028	0.000
SBD	0.097	0.152	0.002	-0.002	0.001		0.004	0.000	-0.001	-0.002	-0.003	-0.001	0.000	-0.012	0.000
SpH	0.107	0.085	0.001	0.000	-0.004	0.003		0.000	0.001	0.000	-0.001	0.000	0.000	0.007	0.000
TH	0.002	0.198	0.046	-0.002	-0.015	-0.007	0.005		-0.005	0.000	-0.006	0.000	-0.001	-0.052	0.000
STKC	0.030	0.128	-0.002	0.000	0.011	-0.004	0.004	0.000		-0.001	0.000	0.000	0.000	0.027	0.000
LL	-0.005	0.112	-0.003	0.000	0.005	0.032	0.005	0.000	0.006		0.000	-0.003	0.001	0.020	0.000
AAR	0.017	-0.149	-0.027	0.003	0.006	-0.017	-0.007	-0.001	0.000	0.000		0.001	0.002	0.031	-0.001
LA	-0.003	0.095	0.001	-0.001	0.004	0.039	-0.010	0.000	0.005	-0.005	-0.003		0.000	0.015	0.000
Ele	-0.003	0.200	0.023	-0.003	-0.010	-0.007	0.004	0.001	-0.005	0.001	-0.010	0.000		-0.084	0.001
Lati	0.122	-0.223	-0.021	0.003	0.008	-0.009	0.006	-0.001	0.007	-0.001	0.004	0.000	0.002		-0.001
SP	0.002	0.182	0.022	-0.003	-0.003	0.012	-0.025	0.000	-0.004	0.000	-0.006	0.000	-0.002	-0.079	

Longi, Longitude; Lati, Latitude; Ele, Elevation; AAR, annual average rainfall; SP, Soil porosity; SpH, Soil pH; SBD, Soil bulk density; STKC, Soil total potassium content; BD, Basal diameter; CD, Crown diameter; TH, Diameter height; LA, Leaf area; LL, Leaf length; FY, fresh fruit yield.

Table S16. The result of path analysis for EOY/BD of *L. cubeba*

Factors	Path coefficient (EOY/BD - response factors)													
	Direct path coefficient	Indirect path coefficient												
		FY	FY.BD	EOP	STKC	SBD	SpH	LL	CD	LA	Longi	LW	SMaxWHC	EOC
FY	-0.982		0.869	0.896	-0.001	0.003	0.001	0.001	0.001	0.001	-0.001	0.001	0.000	-0.002
FY/BD	0.965	-0.885		0.819	-0.002	0.003	0.001	0.001	0.001	0.001	-0.001	0.001	0.000	-0.001
EOY	0.982	-0.896	0.805		-0.001	0.005	0.003	0.001	0.001	0.001	-0.001	0.001	0.000	0.001
STKC	-0.007	-0.141	0.257	0.175		-0.001	0.000	0.002	0.000	0.001	0.000	0.001	0.000	0.002
SBD	0.020	-0.168	0.160	0.238	0.000		0.000	0.003	0.000	0.003	-0.001	0.004	0.001	0.000
SpH	0.013	-0.094	0.094	0.199	0.000	0.001		0.000	0.000	-0.001	0.000	-0.002	0.000	0.003
LL	0.009	-0.124	0.160	0.158	-0.001	0.007	0.001		0.000	0.006	0.000	0.008	0.000	0.002
CD	0.004	-0.329	0.149	0.356	0.000	0.001	0.000	0.000		0.000	-0.001	0.000	0.000	-0.002
LA	0.007	-0.105	0.129	0.136	-0.001	0.008	-0.001	0.008	0.000		-0.001	0.010	0.000	0.001
Longi	0.003	0.324	-0.216	-0.270	-0.001	-0.008	0.000	-0.001	-0.001	-0.001		-0.002	0.000	0.007
LW	0.010	-0.078	0.105	0.108	-0.001	0.009	-0.002	0.007	0.000	0.007	-0.001		0.000	0.001
SMaxWHC	-0.001	-0.001	-0.028	-0.103	0.000	-0.013	-0.002	-0.003	0.001	-0.002	-0.001	-0.003		-0.005
EOC	0.015	0.145	-0.093	0.050	-0.001	0.001	0.003	0.001	0.000	0.000	0.001	0.001	0.000	

Longi, Longitude; SpH, Soil pH; SBD, Soil bulk density; STKC, Soil total potassium content; SMaxWHC, Soil Maximum water holding capacity; CD, Crown diameter; LA, Leaf area; LL, Leaf length; LW, Leaf width; FY, fresh fruit yield; FY/BD, Fruit yield/ Basal diameter; EOC, Essential oil content; EOY, Essential oil yield.

Table S17. Results on detrended correspondence analysis of environmental factors

Statistic	DCA1	DCA2	DCA3	DCA4
Eigenvalues	0.165	0.054	0.014	0.013
Additive Eigenvalues	0.165	0.049	0.012	0.011
Decorana values	0.183	0.034	0.009	0.008
Axis lengths	1.488	1.149	0.577	0.613

The gradient length of sorting axis were all less than 3, so that the raw data can be used by the redundancy analysis belonging to the linear model.

Table 18. redundancy analysis (RDA) results for Phenotypic characteristics of *L. cubeba* with environmental factors

	RDA1	RDA2	RDA3	RDA4	Total
Eigenvalue	2.343	1.886	0.897	0.588	
Proportion Explained	0.117	0.094	0.045	0.029	79.19
Cumulative Proportion	0.117	0.211	0.256	0.286	
AAT	0.201	-0.223	-0.163	-0.207	
AAMaxT	0.167	-0.076	-0.209	-0.234	
AAMinT	0.275	-0.284	-0.087	-0.136	
AARH	0.304	0.055	0.141	0.394	
AAR	-0.455	-0.174	0.205	-0.162	
STOC	0.070	0.581	-0.189	-0.233	
SpH	-0.090	-0.145	-0.353	-0.598	
STNC	-0.009	0.480	-0.198	-0.247	
STPC	-0.048	0.569	-0.116	-0.504	
STKC	0.011	-0.307	0.445	-0.424	
SWC	0.182	0.579	0.164	0.059	
SBD	0.521	-0.308	0.246	0.085	
SMaxWHC	-0.086	0.581	-0.034	-0.060	
SMinWHC	0.035	0.541	0.080	0.036	
SP	0.522	0.518	0.234	0.033	
Lati	-0.511	-0.702	0.076	0.128	
Longi	-0.798	-0.370	0.063	0.019	
Ele	0.360	0.597	-0.160	-0.006	
Model			0.001 ***		

***, significant in 0.001 level; AAT, Annual average temperature; AAMaxT, Annual average maximum temperature; AAMinT, annual average minimum temperature; AAR, annual average rainfall; AARH, annual average relative humidity; SWC, Soil water content; SBD, Soil bulk density; SP, Soil porosity; SMaxWHC, Soil Maximum water holding capacity; SMinWHC, Soil Minimum water holding capacity; STOC, Soil total carbon content; SpH, Soil pH; STNC, Soil total nitrogen content; STPC, Soil total phosphorus content; STKC, Soil total potassium content.
